# *Escherichia coli* Global Gene Expression in Urine from Women with Urinary Tract Infection

**DOI:** 10.1371/journal.ppat.1001187

**Published:** 2010-11-11

**Authors:** Erin C. Hagan, Amanda L. Lloyd, David A. Rasko, Gary J. Faerber, Harry L. T. Mobley

**Affiliations:** 1 Department of Microbiology and Immunology, University of Michigan Medical School, Ann Arbor, Michigan, United States of America; 2 Institute for Genome Sciences and Department of Microbiology & Immunology, University of Maryland School of Medicine, Baltimore, Maryland, United States of America; 3 Department of Urology, University of Michigan Medical School, Ann Arbor, Michigan, United States of America; Dartmouth Medical School, United States of America

## Abstract

Murine models of urinary tract infection (UTI) have provided substantial data identifying uropathogenic *E. coli* (UPEC) virulence factors and assessing their expression *in vivo*. However, it is unclear how gene expression in these animal models compares to UPEC gene expression during UTI in humans. To address this, we used a UPEC strain CFT073-specific microarray to measure global gene expression in eight *E. coli* isolates monitored directly from the urine of eight women presenting at a clinic with bacteriuria. The resulting gene expression profiles were compared to those of the same *E. coli* isolates cultured statically to exponential phase in pooled, sterilized human urine *ex vivo*. Known fitness factors, including iron acquisition and peptide transport systems, were highly expressed during human UTI and support a model in which UPEC replicates rapidly *in vivo*. While these findings were often consistent with previous data obtained from the murine UTI model, host-specific differences were observed. Most strikingly, expression of type 1 fimbrial genes, which are among the most highly expressed genes during murine experimental UTI and encode an essential virulence factor for this experimental model, was undetectable in six of the eight *E. coli* strains from women with UTI. Despite the lack of type 1 fimbrial expression in the urine samples, these *E. coli* isolates were generally capable of expressing type 1 fimbriae *in vitro* and highly upregulated *fimA* upon experimental murine infection. The findings presented here provide insight into the metabolic and pathogenic profile of UPEC in urine from women with UTI and represent the first transcriptome analysis for any pathogenic *E. coli* during a naturally occurring infection in humans.

## Author Summary

Animal models of infection have been used extensively to study how bacteria and other pathogens cause disease. These models provide valuable information and have led to the development of numerous vaccines and antimicrobial therapies. However, it is important to recognize how these animal models compare to human infection and to understand how bacteria cause disease in humans. This study measured gene expression in *E. coli*, a major cause of urinary tract infection, immediately after collection from the urine of women with bladder infection symptoms. The data showed that *E. coli* gene expression in the urine from women with urinary tract infection was very often similar to what had been observed in a mouse model, but these studies also identified several potentially important differences, including a bacterial surface structure that is necessary for infection in mice but not detected in most *E. coli* in human urine. Although more precise measurements are still needed, these findings contribute to our understanding of bacterial infection in humans and will help in the development of vaccines and treatments for urinary tract infection.

## Introduction

Animal models of infection have provided valuable insight into diverse mechanisms of bacterial pathogenesis. Application of microarray technology to these models has further enabled analysis of bacterial global gene expression during infection of a specific host. These studies have included transcriptional profiles of pathogenic *Escherichia coli* in macrophages [1], host epithelial cells [2], and mice [3], [4]. More recently, a limited number of groups have measured genome-wide expression of bacterial pathogens during infections of a human host, including *Vibrio cholerae* in rice water stool of cholera patients [5], [6], *Pseudomonas aeruginosa* in sputum from cystic fibrosis patients [7], and *M. tuberculosis* in resected lung specimens [8]. When these data were compared to results of animal model transcriptional studies, host-specific differences were observed [8].

The urinary tract is among the most common sites of bacterial infection in humans, and *E. coli* is by far the most common species infecting this site, accounting for more than 80% of community-acquired infections [9]. Uncomplicated UTIs include cystitis infections in adult women who are not pregnant and do not suffer from structural or neurological dysfunction [10]. Cystitis, a clinical diagnosis presumed to represent infection of the bladder, is defined by the presence of ≥10^3^ bacteria/ml in a midstream, clean-catch urine sample from a patient with symptoms including dysuria, urinary urgency, and increased frequency [11], [12]. Forty percent of adult women will experience symptoms of cystitis during their lifetime and there is a 25% risk that a second symptomatic episode will occur within 6–12 months [13].

Uropathogenic *E. coli* (UPEC) represent a specific subset of *E. coli* capable of colonizing the urinary tract and eliciting the symptoms of cystitis and pyelonephritis. Genetically distinct from commensal *E. coli* found in the intestinal tract, these strains contain numerous genomic insertions into the “backbone” *E. coli* chromosome, both as pathogenicity-associated islands (PAIs) [14], [15], [16] and shorter islet sequences. In pyelonephritis isolate CFT073, for example, genomic islands and islets comprise over 20% of the genome [14]. Acquired by horizontal gene transfer, PAIs often encode proteins that contribute to pathogenesis; loss of these regions may attenuate virulence [17], [18].

An array of virulence and fitness factors has been described that allow UPEC to access and persist in the urinary tract niche. Flagellin-dependent motility is required for ascension to the kidneys [19] and secreted toxins including hemolysin, cytotoxic necrotizing factor 1, and secreted autotransporter toxin elicit damage to the host epithelium [20], [21], [22]. Polysaccharide capsule [23] and immunosuppressive proteins [24] also contribute to urinary tract colonization and may allow immune evasion. Finally, as the urinary tract represents a unique nutritional niche, TonB-dependent metal acquisition systems are required for UPEC survival in this iron-limited environment [25] and recent evidence suggests that these pathogens metabolize peptides and amino acids as a primary carbon source [26]. Transcriptome analysis of strain CFT073 during murine experimental UTI demonstrated that many of these fitness factors are upregulated during infection [4].

Perhaps the most well-defined UPEC virulence factors are type 1 fimbriae, adhesive structures required for complete colonization of the murine urinary tract [27], [28], [29]. Encoded by virtually all *E. coli* strains, type 1 fimbriae mediate urinary tract adherence via the FimH fimbrial tip adhesin, which binds to mannosylated uroplakins located on the uroepithelium surface [30]. This interaction elicits a host response, including induction of pro-apoptotic and epithelial differentiation factors [31], as well as secretion of the pro-inflammatory cytokines interleukin-6 and IL-8 [32]. Expression of type 1 fimbriae is phase variable, controlled by an invertible DNA element that contains the promoter for the major structural subunit gene, *fimA* [33]. During murine experimental UTI, *fimA* was the fourth most highly-expressed gene and the other *fim* operon genes were also upregulated *in vivo* as compared to *in vitro* culture [4].

To date, much of the work in this field has used *E. coli* strains isolated from patients with UTI. The investigation of UPEC pathogenesis, however, has largely been conducted using *in vitro* models and the well established murine model of ascending UTI [34]. Volunteer colonization studies utilizing a non-pathogenic asymptomatic bacteriuria strain have shed light into mechanisms that promote *E. coli* survival within the human urinary tract [35], [36], but do not represent UTIs caused by *E. coli* with full pathogenic potential. If we wish to fill gaps in our understanding of this widespread human pathogen, it is crucial to focus on UPEC gene expression during naturally-occurring human UTI.

In this study, we measure gene expression in *E. coli* isolated immediately following collection from the urine of eight women experiencing symptoms of UTI. The data presented provide insight into the metabolic and virulence profile of multiple UPEC strains during human infection. We propose that *E. coli* utilizes an array of iron acquisition systems and has access to plentiful carbon sources during human UTI, allowing for robust replication and may downregulate or transiently express a major virulence factor, type 1 fimbria.

## Results

### Transcriptome analysis of *E. coli* UTI in urine from women with UTI

Thirty-six urine samples were collected from 34 female patients (ages 21–89, mean = 47) attending a urology clinic with presumptive bacteriuria. Nineteen urine samples were culture-negative and six specimens were culture-positive for bacterial species other than *E. coli*, including coagulase-negative *Staphylococcus* sp. (*n* = 2), *Acinetobacter baumannii* (*n* = 1), *Enterobacter cloacae* (*n* = 1), *Enterococcus* sp. (*n* = 1), and *Klebsiella pneumoniae* (*n* = 1). Eleven women were culture-positive for *E. coli* and 10 of these specimens were suitable for our study, as poor RNA yield prevented analysis of one *E. coli* specimen. Of these ten *E. coli*-positive samples, two urine specimens contained mixed infections of two different *E. coli* strains. O and H serotyping conducted on these 12 *E. coli* isolates indicated that O6 and O25, which are frequently associated with UTI isolates [37], were the most common serogroups, representing 7 of 12 strains (Table S1). Two isolates (121 and 361), obtained from the same patient on separate clinic visits (5 months apart), had identical serotypes, but could not be conclusively identified as the same strain. Antibiotic susceptibility testing indicated that these clinical isolates also showed high frequencies (up to 7 of 12 isolates) of resistance to common UTI therapies, including trimethoprim-sulfamethoxazole (Table S2).

Of the eight isolates ultimately used in our study (see below), all were collected from pyuria-positive patients who were not catheterized at the time of collection and 6 of 8 were collected from patients reporting a previous UTI. Although these isolates were obtained from urology patients, some with histories of UTI or kidney stones, extensive genotype analysis compared against a collection of over 300 *E. coli* isolates indicated that the virulence gene profiles of these strains most closely matched other cystitis isolates and not fecal-commensal *E. coli* (P. Vigil and H. Mobley, in preparation).

CFT073-specific microarrays were used to measure transcript levels from the eight *E. coli* isolates obtained from single-strain infections, both immediately from the urine of infected women and following static culture to mid-exponential phase in pooled human urine *ex vivo*. Under each condition, 20–30% of all genes measured (5379 ORFs) were classified as expressed for each isolate (Table S3). It is important to note that genes classified as not expressed may be absent or divergent in these strains.

### UPEC replicates rapidly during human UTI

In all strains, the most highly expressed genes in urine from women with UTI were those encoding ribosomal subunits. Indeed, ribosomal genes comprised 24–54% of the top 50 most highly expressed genes in each clinical isolate. It is well established that *E. coli* rRNA and ribosomal protein mRNA synthesis increases proportionally to growth rate [38], [39]. Consistent with this, the most highly expressed non-ribosomal genes also suggest rapid bacterial growth *in vivo* (Table 1). Genes encoding transcription and translation machinery (*infC*, *yfiA*, *rpoA*, *rhoI*, *tufA*, *fusA*, *tufB*, *efp*), F_0_F_1_ ATPase components (*atpE*, *atpF*), fatty acid biosynthesis factors (*acpP*, *fabI*), protein folding and secretion apparatus (*slyD*, *secG*, *prlA*), and outer membrane components (*ompA*) were among the most highly expressed during human infection.

**Table 1:** Genes among the top 50 non-ribosomal genes expressed by at least half of clinical isolates *in vivo* a.

Gene	Annotation	Median rank^b^	No. strains^c^ (*n* = 8)
*tufB*	Elongation factor Tu	8	8
*rpoA*	DNA-directed RNA polymerase alpha chain	17	8
*prlA*	Preprotein translocase SecY subunit	22	7
*tufA*	Elongation factor Tu	23	8
*fusA*	Elongation factor G	26	8
*acpP*	Acyl carrier protein	34	8
*yfjA*	16S rRNA processing protein RimM	36	8
c1363	Hypothetical protein	38	8
*atpE*	ATP synthase C chain	46	8
*infC*	Translation initiation factor IF-3	49	8
*ompA*	Outer membrane protein A precursor	53	8
c2114	Hypothetical protein	56	8
c0693	Hypothetical protein	64	6
*rhoL*	Very hypothetical rho operon leader peptide	70	7
*aceE*	Pyruvate dehydrogenase E1 component	71	6
*cspA*	Cold shock protein A	71	5
*secG*	Protein-export membrane protein	71	6
*yceD*	Hypothetical protein	78	5
*lpdA*	Dihydrolipoamide dehydrogenase	78	7
*gapA*	Glyceraldehyde 3-phosphate dehydrogenase A	80	5
*trmD*	tRNA (Guanine-N(1)-)-methyltransferase	80	5
*ptsH*	Phosphocarrier protein HPr	80	6
*tpiA*	Triosephosphate isomerase	81	6
*ahpC*	Alkyl hydroperoxide reductase C22 protein	82	5
*glnA*	Glutamine synthetase	83	5
c1040	Hypothetical protein	86	5
*efp*	Elongation factor P	86	5
*ydgR*	Putative tripeptide transporter permease TppB	89	5
*atpF*	ATP synthase B chain	94	6
c4663	Hypothetical protein	96	4
*yihK*	GTP-binding protein typA/BipA	96	5
*yhdG*	tRNA-dihydrouridine synthase B	99	4
*eno*	Enolase	100	4
*cydA*	Cytochrome D ubiquinol oxidase subunit I	102	4
c4661	Hypothetical protein	102	4
*slyD*	FKBP-type peptidyl-prolyl cis-trans isomerase	104	4
*fis*	DNA-binding protein	104	4
*fba*	Fructose-bisphosphate aldolase class II	105	4
*rho*	Transcription termination factor Rho	105	4
*fabI*	Enoyl-[acyl-carrier-protein] reductase [NADH]	110	4
*ackA*	Acetate kinase	119	4
c4889	Hypothetical protein	144	4
*chuA*	Outer membrane heme/hemoglobin receptor	205	4

a Genes encoding ribosomal subunits were excluded and only genes expressed by ≥4 strains are listed.

b Gene expression rank (of 5379 ORFs) when genes listed in order of microarray signal intensity.

c Number of strains for which indicated gene is among the top 50 non-ribosomal genes expressed *in vivo*. Gene may be expressed by other isolates (but not among top 50).

Because variable host nucleic acid present in the *in vivo* samples prevented direct comparison between *in vivo* and *in vitro* conditions, a more conservative, relative comparison was derived. To identify expression differences between *E. coli* growth in urine during cystitis and growth in urine *ex vivo*, genes were ranked in order of microarray signal intensity, yielding an estimate of relative gene expression. For each isolate, these rank values were compared between *in vivo* and *in vitro* conditions. Genes for which the expression rank significantly changed between these conditions were considered differentially expressed.

Although we noted that two cold-shock-associated genes, *cspA* and *deaD*, were upregulated in human urine samples (Table 2), expression of *cspA* is known to occur during non-cold shock stress conditions [40], [41]. Moreover, both *deaD* and *cspA* were also upregulated by UPEC [4] and an ABU strain [35] following expulsion from the murine and human bladders, respectively. Nevertheless, to assess the effect of temperature downshift that may have occurred during sample processing, transcript levels of a representative virulence gene, *fimA*, were measured after a urine culture of strain CFT073 was moved from 37°C to room temperature (Fig. S1). *fimA* transcript levels did not significantly change after 10 min (or up to 60 min) at room temperature, suggesting that sample processing was not responsible for these results.

**Table 2:** Genes upregulated *in vivo* compared to growth in human urine *in vitro*.

Gene	Annotation	Median Δ rank^a^	No. strains^b^ (*n* = 8)
*cspA*	Cold shock protein A	1260	8
c4376	Hypothetical protein (overlaps *cspA* on antisense strand)	1234	7
*yhfC*	Uncharacterized transporter of major facilitator superfamily	1123	7
*fis*	DNA-binding protein	1102	7
*suhB*	Inositol-1-monophosphatase	1098	7
*gltP*	Proton glutamate symport protein	1097	7
c5293	Hypothetical protein (overlaps *rplI* on antisense strand)	1076	6
*ydgR*	Putative tripeptide transporter permease TppB	1071	8
*yifK*	Probable amino acid or metabolite transport protein	1070	6
*ndh*	NADH dehydrogenase II	1060	8
*rplQ*	50S ribosomal protein L17	1032	7
*tgt*	Queuine tRNA-ribosyltransferase	1020	8
*rnpA*	Ribonuclease P protein component	1009	8
*deaD*	Cold-shock DEAD-box protein A	1000	7
*yidC*	Putative inner membrane protein translocase component	967	8
*guaB*	Inosine-5′-monophosphate dehydrogenase	963	7
*ptsG*	PTS system, glucose-specific IIBC component	922	6
*yhdG*	tRNA-dihydrouridine synthase B	911	8
*pyrH*	Uridylate kinase	908	7
*brnQ*	Branched-chain amino acid transport system II carrier protein	903	7
*secF*	Protein-export membrane protein	884	7
*glnP*	Glutamine transport system permease protein	874	8
*secD*	Protein-export membrane protein	869	6
*carA*	Carbamoyl-phosphate synthase small chain	857	6
*gidA*	Glucose inhibited division protein A	852	7
*ychF*	GTP-dependent nucleic acid-binding protein EngD	849	7
*ybeA*	rRNA large subunit methyltransferase	840	6
*guaA*	GMP synthase [glutamine-hydrolyzing]	838	6
*rfe*	Undecaprenyl-phosphatealpha-N-acetylglucosaminyltransferase	835	6
*ybeB*	Hypothetical protein	819	7
*ftsW*	Cell division protein FtsW	795	6
*fldB*	Flavodoxin 2	795	6
*yabB*	Cell division protein MraZ	792	7
*yjfG*	UDP-N-acetylmuramate:L-alanyl-gamma-D-lutamyl-meso-diaminopimelate ligase	786	7
*tig*	Trigger factor	785	7
*pyrG*	CTP synthase	773	6
*speE*	Spermidine synthase	761	6
*yhbC*	Ribosome maturation protein RimP	758	8
*truB*	tRNA pseudouridine synthase B	757	6
c0526	Hypothetical protein	757	6
*yjgP*	LPS transporter inner membrane component LptF	741	6
*ycfB*	tRNA(5-methylaminomethyl-2-thiouridylate)-methyltransferase	738	7
*gltX*	Glutamyl-tRNA synthetase	719	6
c2839	Hypothetical protein (overlaps *pta* on antisense strand)	716	6
*lepA*	GTP-binding elongation factor	715	6
*valS*	Valyl-tRNA synthetase	690	6
*pta*	Phosphate acetyltransferase	679	6
*yqiA*	Esterase	634	6

a Median change in relative expression rank (genes ranked in order of expression; highest, 1; lowest, 5379) *in vitro* compared to *in vivo.*

b Number of strains for which indicated gene was among the 250 (4.6%) most upregulated genes. Gene may be expressed/upregulated by other strains, but was not among the top 4.6%.

Cell division factors (*ftsW*, *mraZ*), tRNA processing and translation machinery (*rnpA*, *tgt*, *rplQ*, *rimP*, *truB*, *valS*, *gltX*, *yhdG*, *ybeA*, *ycfB*, *lepA*, *ychf*), and protein secretion components (*secD*, *secF*, *yidC*) were among genes upregulated *in vivo* by the majority of strains, suggesting that *E. coli* may be replicating faster in the human urinary tract than *in vitro* (Table 2). Similarly, genes involved in nucleotide synthesis (*guaA*, *guaB*, *pyrG*, *pyrH*), lipopolysaccharide assembly (*lptF*), peptidoglycan recycling (*yjfG*), and enterobacterial common antigen synthesis (*rfe*) were also upregulated. Taken together, these data indicate that *E. coli* was likely replicating very rapidly during symptomatic UTI in women.

### Microarray correlates with qPCR

**Figure 1:**
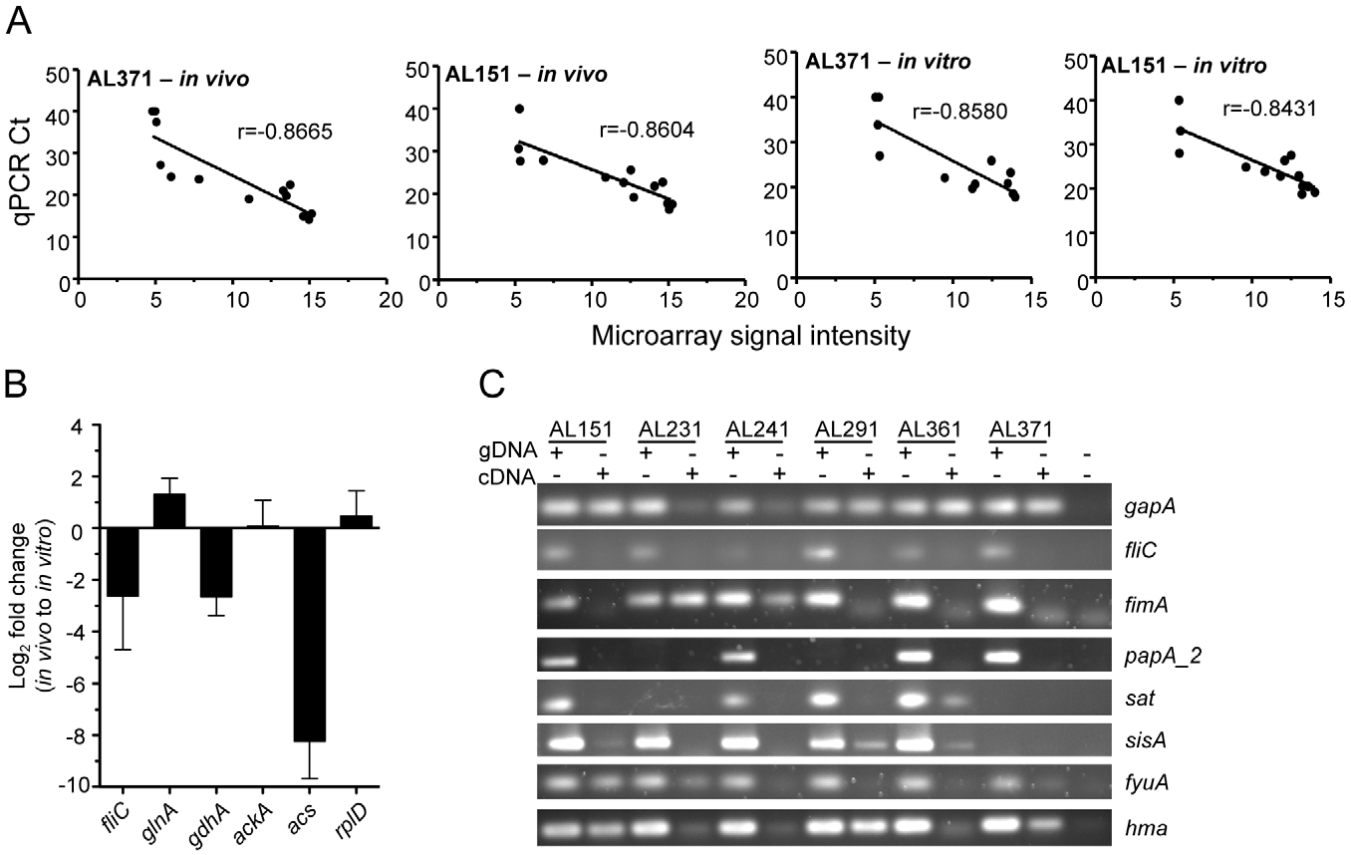
*E. coli* gene expression in voided urine from cystitis patients and culture in urine *ex vivo*. (A) Correlation of gene expression levels obtained by microarray and qPCR. Ct values determined by qPCR are plotted for 13 genes (see text) *versus* normalized microarray signal intensity for *in vivo* (left two panels) and *in vitro* (right two panels) cDNA samples from patient isolates 371 (first and third panel) and 151 (second and last panel). Correlation coefficient (r) values are shown and *P*<0.001 for all correlations. (B) Genes differentially expressed during UTI in women and culture in urine *ex vivo*. Log_2_ fold changes *in vivo*, compared to *in vitro* expression, were measured by qPCR and are normalized to *gapA* transcript. (C) Confirmation of *in vivo* gene expression by clinical isolates as shown by PCR using genomic DNA or cDNA template.

To confirm these microarray findings, qPCR was performed using *in vivo*- and *in vitro*-derived cDNA templates of two representative *E. coli* strains isolated in this study, AL151 and AL371. Strains were selected based on availability of *in vivo* cDNA. The threshold cycle (Ct) values of the 13 genes measured (*gapA*, *hma*, *fyuA*, *fimA*, *papA_2*, *fliC*, *sat*, *sisA*, *glnA*, *gdhA*, *ackA*, *acs*, and *rplD*) correlated (*P*<0.001 or r<−0.8431) with the normalized microarray signal intensities of each gene (Fig. 1A). Similarly, genes identified as differentially expressed between *in vivo* and *in vitro* conditions were consistently found to be up- or down-regulated in these two strains by qPCR measurement (Fig. 1B). Finally, PCR using *in vivo*-derived cDNA generally confirmed expression of select virulence genes (Fig. 1C).

### *E. coli* undergoes aerobic or microaerobic respiration during human UTI

Genes involved in aerobic respiration tended to be highly expressed by most strains assessed immediately after expulsion from the human bladder. Encoding a component of cytochrome *d* complex, which is maximally expressed during microaerobiosis, *cydA* was among the top 50 most expressed non-ribosomal genes by four isolates (Table 1) and was in the top 3% of genes expressed by all strains *in vivo* and was similarly expressed during urine culture *in vitro*. Expression of the cytochrome *o* oxidase genes *cyoABCDE*, which are expressed in oxygen-rich conditions, varied among patients. These genes were strongly expressed by 5 of 8 strains *in vivo* (top 7%), more weakly expressed by 2 of 8 strains (top 20–30%), and just below background in one strain. This is in contrast to a recent *E. coli* transcriptome study for an asymptomatic bacteriuria strain, which noted consistent downregulation of *cyoABCD* during intentional colonization of the human bladder [35]. For all strains, expression of *cyoABCD* and *cydAB* did not appear to differ between *in vivo* and *in vitro* samples or from expression by *E. coli* CFT073 during experimental infection of the murine urinary tract [4], suggesting similar oxygenation in these conditions.

Genes that encode a terminal electron acceptor pathway used during anaerobiosis were not uniformly expressed by all strains. For example, the genes encoding nitrate reductase I, *narGHJIK*, were only expressed *in vivo* by two strains, AL121 and AL151. Formate dehydrogenase N (*fdnGHI*), induced by nitrate [42] and anaerobic conditions [43], was expressed by *E. coli* in half of UTI patients (isolates AL121, AL151, AL291, and AL361). Nitrite-inducible genes *nirBDC* were also expressed in half of patients (isolates AL121, AL151, AL291, and AL371). Interestingly, the three patient isolates that only weakly expressed *cyoABCDE* also expressed nitrate reductase, formate dehydrogenase N, and nitrite reductase, suggesting that these isolates likely experienced a more anaerobic and nitrate/nitrite-rich environment and may be using these products for anaerobic respiration. Together, these data indicate that oxygen and/or nitrate levels in voided urine vary by patient and suggest that pathogenic *E. coli* adapts to utilize either form of respiration.

### *E. coli* experiences nitrogen limitation *in vivo*

Previous transcriptomic analysis of *E. coli* indicated that the murine urinary tract is nitrogen-limiting for this pathogen, despite a high urea concentration in urine [4]. Glutamine synthetase (*glnA*), which assimilates ammonia with high affinity in an energy-dependent manner and is transcriptionally induced by nitrogen-limited growth [44], was among the most highly expressed genes in urine from 5 of 8 cystitis patients (Table 1). By qPCR, *glnA* was upregulated *in vivo* (2.9-fold) in the two strains tested (AL151 and AL371), while the low affinity energy-independent glutamate dehydrogenase *gdhA*, which is transcriptionally repressed in low nitrogen conditions [45], was downregulated 8.3-fold (Fig. 1B). This indicates that *E. coli* similarly experiences nitrogen limitation during infection of the human urinary tract. High concentrations of nitrogen in urea are not available to the urease-negative *E. coli*.

### Carbon metabolism suggests abundant carbon sources *in vivo*

The gene encoding acetyl-CoA synthetase, *acs*, was one of the most strongly downregulated genes in all *E. coli* strains during growth in urine from women with UTI as compared to culture in urine *ex vivo* (Table 3). Involved in acetate assimilation by conversion to pyruvate, expression of this enzyme is activated by decreasing oxygen and increasing cAMP levels [46]. In contrast, genes involved in acetate excretion, shown to contribute to urovirulence in a murine model [47], were strongly expressed *in vivo*. The phosphate acetyltransferase (*pta*) gene was among the top 100 genes upregulated *in vivo* in 6 of 8 isolates (Table 2) and acetate kinase (*ackA*) was one of the most highly expressed non-ribosomal genes in 50% of the strains (Table 1). By qPCR, *acs* was downregulated nearly 600-fold *in vivo*, while *ackA* was relatively unchanged (Fig. 1B). Acetate-induced genes [48] *glcBGD* and the glyoxylate shunt genes *aceAB* were also downregulated (Table 3). Furthermore, components of the pyruvate dehydrogenase complex, which functions upstream of AckA-Pta to convert pyruvate to acetyl-CoA, *aceE* and *lpdA* were among the most highly *in vivo* expressed genes in the majority of patient isolates (Table 1). These expression patterns imply that *E. coli* produces, but does not assimilate acetate during growth in the human bladder.

**Table 3:** Genes downregulated *in vivo* compared to growth in human urine *in vitro*.

Gene^a^	Annotation	Median Δ rank^b^	No. strains^c^ (*n* = 8)
*glcB*	Malate synthase G	−1730	7
*glcG*	Protein glcG	−1715	7
*osmC*	Osmotically inducible protein C	−1705	8
*glcD*	Glycolate oxidase subunit	−1700	8
*ilvB*	Acetolactate synthase isozyme I large subunit	−1673	7
c2623	Fructose-bisphosphate aldolase class I	−1647	8
*osmY*	Osmotically inducible protein Y precursor	−1637	8
*aceB*	Malate synthase A	−1634	8
*yjcG*	Putative symporter	−1622	8
*ygjG*	Probable ornithine aminotransferase	−1619	8
*amyA*	Cytoplasmic alpha-amylase	−1608	8
*phoH*	PhoH protein	−1603	8
*aceA*	Isocitrate lyase	−1585	7
*rpsV*	30S ribosomal protein S22	−1563	8
*leuD*	3-isopropylmalate dehydratase small subunit	−1553	7
*aldB*	Aldehyde dehydrogenase B	−1550	8
*aldA*	Aldehyde dehydrogenase A	−1550	8
*mglB*	D-galactose-binding periplasmic protein precursor	−1548	8
*acs*	Acetyl-coenzyme A synthetase	−1548	8
*leuC*	3-isopropylmalate dehydratase large subunit	−1545	8
*leuA*	2-isopropylmalate synthase	−1526	8
*wrbA*	Flavoprotein	−1519	8
*yiaK*	Hypothetical oxidoreductase	−1512	7
*msyB*	Acidic protein msyB	−1500	8
*elaB*	ElaB protein	−1488	8
*yhiO*	Universal stress protein B	−1481	7
*adhP*	Alcohol dehydrogenase, propanol-preferring	−1459	7
*dadA*	D-amino acid dehydrogenase small subunit	−1449	7
*ecnB*	Putative toxin of osmotically regulated toxin-antitoxin	−1443	8
*sgbU*	Putative hexulose-6-phosphate isomerase	−1436	7
*potF*	Putrescine-binding periplasmic protein precursor	−1429	7
*sbmC*	DNA gyrase inhibitory protein	−1400	8
*talA*	Transaldolase A	−1399	7
*leuB*	3-isopropylmalate dehydrogenase	−1397	7
*ppsA*	Phosphoenolpyruvate synthase	−1378	8
*fumA*	Fumarate hydratase class I, aerobic	−1372	7
*acnA*	Aconitate hydratase 1	−1364	8
*kgtP*	Alpha-ketoglutarate permease	−1363	8
*potH*	Putrescine transport system permease protein	−1360	7
*rspB*	Starvation sensing protein	−1350	7
*ytfQ*	ABC transporter periplasmic binding protein	−1347	7
*hdhA*	7-alpha-hydroxysteroid dehydrogenase	−1347	8
*treA*	Periplasmic trehalase precursor	−1347	7
c1843	Putative glyceraldehyde 3-phosphate dehydrogenase C	−1321	7
*araH*	High-affinity L-arabinose transport system	−1247	8
*sdhB*	Succinate dehydrogenase iron-sulfur protein	−1241	7
*bolA*	BolA protein	−1239	7
*yqeF*	Probable acetyl-CoA acetyltransferase	−1232	7
c3746	2,5-diketo-D-gluconic acid reductase A	−1223	8
*ybaT*	Hypothetical transport protein	−1202	7
*ybaS*	Probable glutaminase	−1184	7
*cbpA*	Curved DNA-binding protein	−1048	8
*cspD*	Cold shock-like protein	−941	7

a Genes with annotated functions are shown. Downregulated hypothetical genes are shown in Table S4.

b Median change in relative expression rank (genes ranked in order of expression; highest, 1; lowest, 5379) *in vitro* compared to *in vivo*.

c Number of strains for which indicated gene was among the 250 (4.6%) most downregulated genes. Gene may be downregulated by other strains, but was not among the top 4.6%.

In bacteria, the acetate switch occurs when cells transition from acetate dissimilation to assimilation. The expression of dissimilatory genes by *E. coli* during infection of the human urinary tract indicates not only that the bacteria have not yet reached stationary phase *in vivo*, but also that they may be utilizing acetogenic carbon sources. Acetogenesis occurs during mixed acid fermentation under anaerobic conditions or aerobically when rapid growth on excess carbon sources limits flux through the tricarboxylic acid (TCA) cycle by excessive NADH production (reviewed in [49]). Because data presented here support rapid aerobic or microaerobic growth of *E. coli* during human UTI, this suggests that the observed acetogenesis is due to “overflow metabolism” and not mixed acid fermentation. This further implies that easily assimilable carbon sources are available to UPEC during symptomatic UTI.

**Figure 2:**
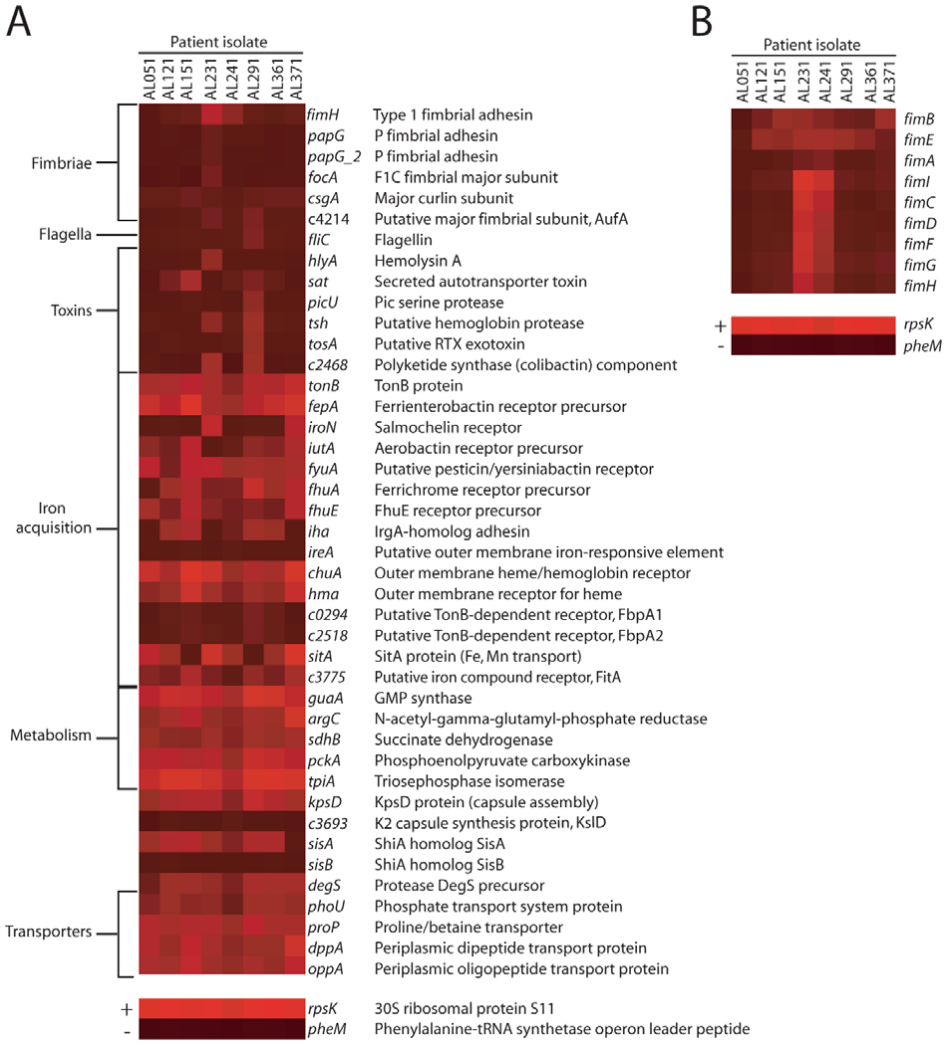
*E. coli* virulence factor expression in urines from UTI patients. Heat maps indicate normalized microarray signal intensities for genes encoding (A) UPEC fitness factors and (B) genes in the *fim* locus in eight *E. coli* isolates in urine collected from women with cystitis. For reference, the overall most (*rpsK*, +) and least (*pheM*, −) expressed genes are shown in the bottom panels, representing average signal intensities of 15.821 and 3.881, respectively.

Peptides and amino acids have been previously implicated as primary carbon sources for UPEC during colonization of the murine urinary tract [26]. Di- and oligopeptide transport components *dppA* and *oppA*, shown previously to contribute to UPEC virulence [26], were strongly expressed by all strains during UTI in women (Fig. 2A). Additionally, a putative tripeptide transporter, *ygdR* (*tppB*), was upregulated *in vivo* by all *E. coli* isolates (Table 2) and was among the top 50 most highly expressed non-ribosomal genes in 5 of 8 strains (Table 1). Other amino acid or metabolite transporters, *yifK*, *yhfC*, and *brnQ* were also upregulated *in vivo*. These findings suggest that *E. coli* may use peptides as an energy source during human UTI, as well.

When compared to culture in urine *ex vivo*, *E. coli* in voided urine from UTI patients downregulated a number of genes involved in metabolic functions (Table 3). The *talA* gene, which encodes one of two transaldolases in the *E. coli* pentose phosphate pathway and does not to contribute to UPEC fitness in a murine model [26], was downregulated. Consistent with rapid growth and TCA cycle saturation, genes in this pathway (*sdhB*, *acnA*, and *fumA*) as well as α-ketoglutarate permease (*kgtP*), which imports a TCA cycle intermediate, were also downregulated. Indeed, growth on excess glucose (an easily assimilable carbon source) has long been appreciated to inhibit expression of *sdhAB* [50] and the succinyl-CoA synthetase complex (reviewed in [49]). Overall, dehydrogenase-type enzymes were frequently downregulated *in vivo* compared to growth *in vitro*, consistent with the hypothesis that rapid growth *in vivo* requires *E. coli* to adapt by modifying the TCA cycle to optimally regenerate NAD^+^. Together, the finding that genes involved in acetogenic growth, peptide import, and the TCA cycle are expressed or modulated during human cystitis is consistent with the requirement of these pathways for UPEC virulence and support the current model of UPEC metabolism during UTI [26], [47].

### Virulence gene expression *in vivo*

While a number of studies have assessed expression of UPEC virulence genes during experimental infection of animal models [3], [4], [51], much less is known about expression patterns during human infection. These microarray data show most strikingly that the expression of genes involved in adherence to host tissues that are highly expressed during murine infection [4], was not detected in the majority of patient isolates (Fig. 2A). Genes encoding P, F1C, or Auf fimbriae were not expressed (*i.e*., below background) in any *E. coli* strains after expulsion from the human urinary tract or gene absence/divergence otherwise prevented detection of these transcripts. Furthermore, the type 1 fimbrial adhesin *fimH*, which is required for virulence during murine infection [27], [28] and invasion [29], was only expressed at detectable levels by *E. coli* in 2 of 8 patients. These strains, AL231 and AL241, also expressed the remaining genes in the *fim* locus, while the other six strains did not (Fig. 2B). To validate these microarray findings, PCR was performed on *in vivo*-isolated cDNA from all strains except AL051 and AL121 (for which no *in vivo*-derived cDNA sample remained). This confirmed the expression of the major structural subunit *fimA* only by strains AL231 and AL241, as well as lack of P fimbrial (*papA_2*) gene expression by strains AL151, AL241, AL361, and AL371 (Fig. 1C). Strains AL231 and AL241, which were shown to express *fimA in vivo* by RT-PCR (Fig. 1C), encoded genes that were 90% identical to *fimA*_CFT073_ (Fig. S4). Because *fimA* transcripts were not detected by microarray for these strains (Fig. 2), we can conclude that our microarray hybridization conditions required greater than 90% sequence identity to yield signal significantly above background. PCR results for additional virulence genes were also generally consistent with their expression by microarray (Fig. 1C, Fig. 2A).

Few patient isolates appeared to express toxin-encoding genes with significant sequence identity to CFT073 toxins above background levels *in vivo* (Fig. 2A). Two isolates expressed *sat* or genes encoding the non-ribosomal peptide/polyketide colibactin significantly above background and expression of both colibactin and *tsh* was only detected in a single isolate (strain AL291). Consistent with transcriptome data from murine UTI [4], flagellin (*fliC*) gene expression was downregulated *in vivo*, as compared to *in vitro* culture (Fig. 1B).

As alluded to above, metabolic pathways have also been implicated in UPEC virulence. The central metabolic pathways of gluconeogenesis and the TCA cycle are required for UPEC fitness in the murine urinary tract [26] and *sdhB*, *pckA* and *tpiA*, encoding enzymes in these pathways, although downregulated compared to growth *in vitro*, were nonetheless expressed in all patient isolates (Fig. 2A). Other metabolic genes implicated in urovirulence, including *guaA* and *argC* [52] were also expressed *in vivo* by all *E. coli* strains and *guaA* was upregulated relative to culture in urine *ex vivo* (Table 2). Expression of D-serine dehydratase (*dsdA*), which processes the putative metabolite signal D-serine [53], was detected in half of the bladder-expelled *E. coli* strains measured.

Genes involved in siderophore production and iron acquisition were globally the most highly expressed fitness determinants across all eight isolates following infection of the human urinary tract. All isolates robustly expressed *tonB*, *fepA*, and *fyuA*, although genes for the synthesis of enterobactin and yersiniabactin siderophores were sporadically detected, possibly due to inherently lower transcript levels for these enzymes (Fig. 2A). Nearly all (7 of 8) strains expressed the heme receptor-encoding *chuA* and in half of patients, this gene was among top 50 most highly expressed non-ribosomal genes (Table 1). In contrast, the heme receptor *hma* was expressed above background in just 4 of 8 patient isolates. *In vivo* expression of genes for the salmochelin receptor *iroN* and aerobactin receptor *iutA* was only observed in two isolates, while receptor genes *ireA*, *fpbA1* and *fbpA2* were not expressed by any strains, either *in vivo* or *in vitro*. Despite these differences, all strains were capable of growth *in vitro* under iron-limiting culture conditions in the presence of the chelator 2′2-dipyridyl (200 µM), although strains AL231 and AL241 could not replicate under more stringent iron-limitation (400 µM) (Fig. S2). These data provide further support for the well-established model of the human urinary tract as an iron-limiting environment [4], [25], [35].

Interestingly, *in vivo* and *in vitro* expression of iron uptake systems did not always correlate and isolates occasionally expressed a specific system in only one condition. For example, patient isolates AL121 and AL241 expressed genes coding for yersiniabactin production only during *in vitro* culture, while strain AL231 expressed these genes strictly during *in vivo* conditions (Fig. S3). For strain AL231, poor *in vitro* expression of these and other genes involved in iron acquisition (including *fep*/*ent* and *iro* loci) correlated with the strain's inability to replicate under stringent iron limitation (Fig. S2). This apparent differential expression of iron uptake genes implies that the extent of iron limitation (and by implication, Fur regulation) differs among individual patients, as well as between the human bladder and urine *ex vivo*. Furthermore, it suggests additional regulation of iron acquisition systems during *E. coli* infection; indeed oxygen tension has been shown to affect expression of iron uptake genes by other pathogens [54].

### Pathogen-specific genes expressed during human cystitis

A subset of fitness genes expressed by *E. coli* following infection of the human urinary tract was specific to pathogenic *E. coli*. That is, these genes are absent from the fecal-commensal *E. coli* strain K12, suggesting that they represent horizontally-acquired, putative fitness genes. Iron acquisition components (*fyuA*, *chuA*, *chuS*, *chuW*, *chuX*), capsule synthesis genes (*kpsF*, *kpsD*, *kpsU*, *kpsC*), an inflammatory suppressor (*sisA*), a PAI-associated prophage integrase gene (c2418), and several hypothetical genes were expressed by the majority of UPEC patient isolates *in vivo* (Table 4). These data indicate that the patient isolates examined here express an array of pathogen-specific genes *in vivo* and further imply that they indeed represent pathogenic *E. coli* strains.

**Table 4:** Pathogen-specific^a^ genes expressed in the majority of urines from women with UTI.

Gene	Product/function	No. strains^b^ (*n* = 8)
c0222	Hypothetical protein	7
c2418*	Prophage P4 integrase	7
*fyuA* *	Yersiniabactin receptor precursor	7
c2484*	Hypothetical protein YbdM	6
*sisA*	*shiA*-like inflammation suppressor A	6
c3686*	Hypothetical protein YrbH (KpsF)	7
*kpsD*	Polysialic acid transport protein KpsD	7
c3689	3-deoxy-manno-octolusonate transferase KpsU	6
*kpsC*	Capsule polysaccharide export protein KpsC	6
c4222	Putative DNA processing protein	6
*chuS* *	Putative heme/hemoglobin transport protein	6
*chuA* *	Outer membrane heme/hemoglobin receptor	7
*chuW* *	Putative heme oxygenase	6
*chuX* *	Hypothetical protein	7
*rfaP*	LPS core biosynthesis protein	7
*rfaG*	LPS core biosynthesis protein	7
*rfaQ*	LPS core biosynthesis glycosyltransferase	7
c5060*	Hypothetical protein	6

a Pathogen-specific is defined as genes absent from *E. coli* K12 strain MG1655.

b Number of patient isolates for which the indicated gene is considered expressed (microarray signal intensity is at least four-fold above background).

*Indicates genes present in 10/10 UPEC isolates and 0/3 fecal *E. coli* isolates as determined by comparative genomic hybridization [88].

### Expression of iron uptake and metabolic genes, but not adhesin genes, is similar in voided urine from murine and human UTI

**Figure 3:**
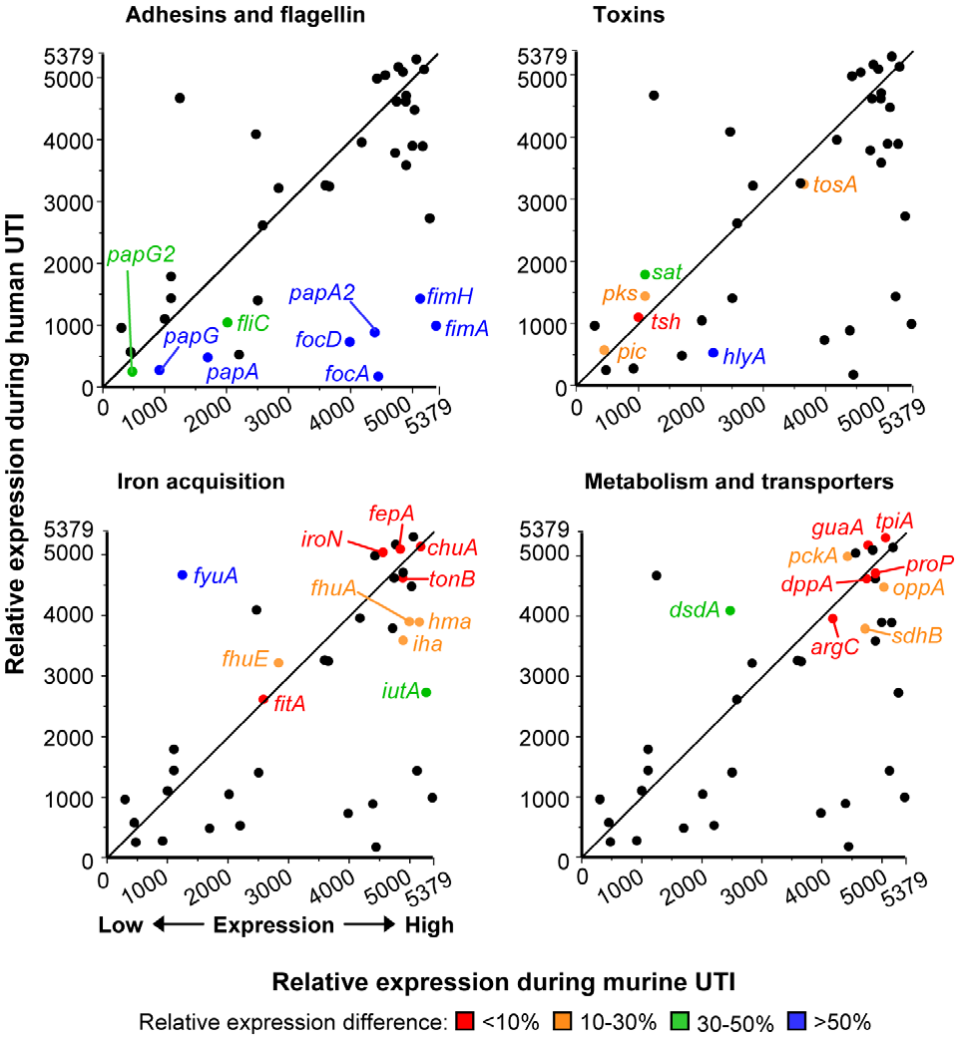
Host-specific virulence gene expression by *E. coli*. Relative virulence and fitness gene expression by *E. coli* isolates in urine from human UTI plotted *versus* relative expression by *E. coli* CFT073 in urine from experimental murine UTI. Line shows the theoretical perfect correlation. Panels highlight fitness genes encoding adherence and motility factors (top left), toxins (top right), iron acquisition systems (bottom left), and metabolic and transport proteins (bottom right). Relative expression is based on normalized microarray signal intensity rank (1, gene with lowest signal; 5379, gene with highest signal). Relative human expression is the median rank of all eight isolates, except for *fimA*, *papA*, *papA_2*, *hlyA*, *sat*, *pic*, *tsh*, *tosA*, *chuA*, *iutA*, *iroN*, *fyuA*, *hma*, *iha*, and *sitA*, which are medians from isolates positive for given gene by PCR (P. Vigil and H. Mobley, in preparation). Highlighted genes are colored to indicate the expression rank difference of each gene between human and murine UTI: red, less than 10% difference in expression rank; orange, 10–30% difference; green, 30–50% difference; blue, greater than 50% difference. Spearman rank correlation coefficient for all genes is r = 0.5890 *(P*<0.0001). Murine data were derived from our previously-published transcriptome study [4].

To compare UPEC virulence gene expression in different mammalian hosts, relative expression levels of 46 fitness genes by CFT073 following experimental murine UTI (derived from [4]) were compared to the average relative expression levels (average expression rank) by *E. coli* patient isolates following human UTI. Overall, relative UPEC virulence gene expression in mice positively correlated with expression in a human host (Spearman r = 0.5890; *P*<0.0001). Genes involved in iron acquisition and metabolism correlated most strongly between the two data sets, with most having less than 10% difference in relative (ranked) expression (Fig. 3). *fyuA* was the exception, due to its poor expression by CFT073 [4], likely attributed to mutations preventing yersiniabactin production [15], [55]. Expression of toxin-encoding genes was moderately similar in expelled urine of the two hosts, differing by only 30–50%. In contrast, adhesin and fimbriae expression was quite different between human and murine urine, with most genes having more than 50% relative expression difference between the two hosts. For example, *fimA* was the fourth most highly expressed gene (relative expression value: 5375) by *E. coli* CFT073 during murine infection, while it had an average rank of 3873 out of 5379 ORFs in women with UTI (relative expression value: 1506).

### Clinical UPEC isolates express type 1 fimbriae *in vitro*

**Figure 4:**
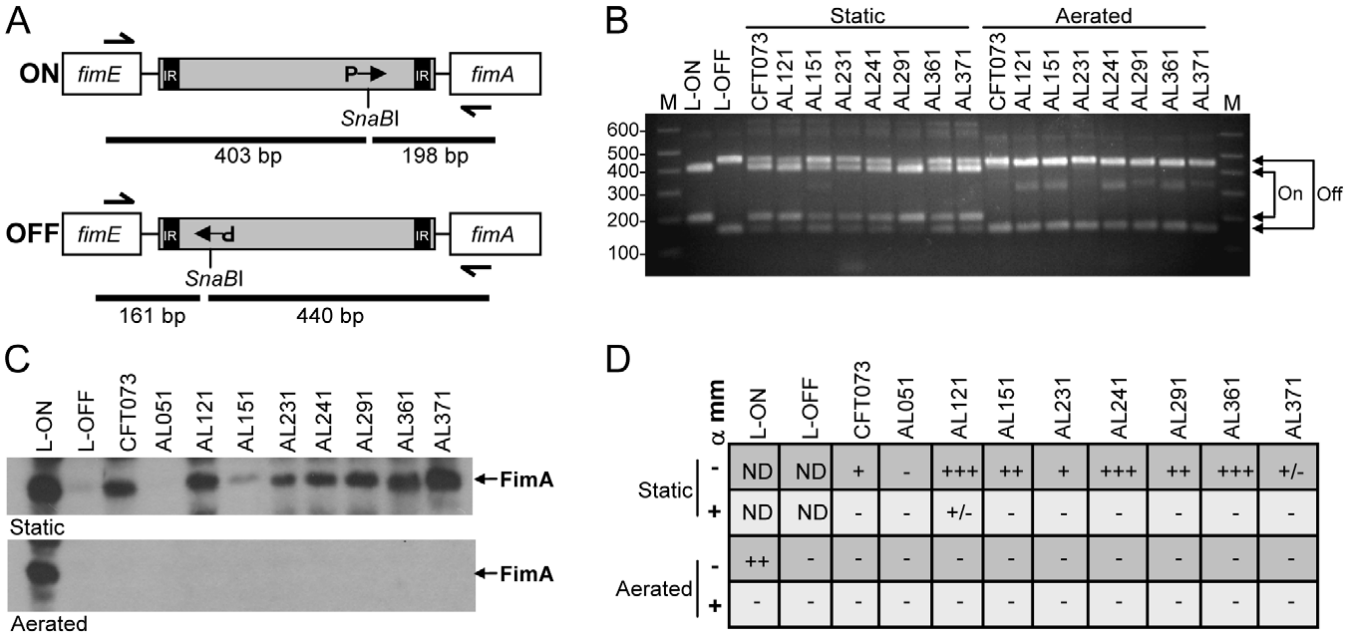
*In vitro* expression of type 1 fimbriae by clinical *E. coli* isolates. (A) Invertible element (IE) assay for type 1 fimbriae [73]. IE is shown in gray; inverted repeats (IR) located within the IE; orientation of the *fim* promoter (P) indicated by arrow in phase-on (top panel) and phase-off (bottom panel) positions. Expected fragment sizes following PCR (half arrows show primer sites) and asymmetrical *SnaB*I digest are indicated by black bars. (B) IE orientation determined as described in (A) for strains cultured statically under type 1 fimbriae-enriching conditions or with aeration. Arrows indicate expected sizes for phase-on and phase-off. CFT073 strains with mutations preventing IE switching and locking the “phase-on” (L-ON) or “phase-off” (L-OFF) orientation are also included. M, molecular mass standards; sizes (in kb) are indicated. (C) Western blot using anti-FimA polyclonal antibody. Strains were cultured as in (B) and acid-treated whole cell lysate separated on 15% SDS-PAGE gels. Top panel, type 1 fimbriae-enriching static culture; bottom panel, aerated culture. Arrows indicate the ∼12 kDa FimA band. (D) Mannose-sensitive hemagglutination. Strains were cultured as in (B) (statically, top two rows; aeration, bottom two rows) and their ability to agglutinate guinea pig erythrocytes in the presence (+) or absence (−) of alpha-methyl-mannoside (α mm) was assessed. −, no agglutination; +/−, weak agglutination; +, agglutination with undiluted or 1∶2 dilution of bacterial suspension; ++, agglutination with 1∶4 or 1∶8 dilution; +++, agglutination with 1∶16 or 1∶32 dilution; ND, not determined. Data represent median agglutination reactions of three independent experiments.

Surprisingly, we found that most (6 of 8) *E. coli* isolates did not express type 1 fimbriae in the urine of patients with UTI. One possible explanation is that these strains do not encode intact type 1 fimbrial genes or that expression of these genes is defective. To distinguish among these possibilities, we examined expression of type 1 fimbriae *in vitro* by the 8 clinical isolates. Using a PCR-based assay (Fig. 4A), we determined the orientation of the *fim* invertible element, the 314 bp region that contains the promoter for the major structural subunit *fimA* and is responsible for phase variation of type 1 fimbriae [33]. As expected, after two 48 h static passages to enrich for type 1 fimbriae expression, all strain populations consisted of bacteria in both the phase-on and phase-off orientations (Fig. 4B). In contrast, when isolates were cultured with aeration for 4 h, only the phase-off orientation could be detected. Invertible element orientations correlated with detection of FimA by western blot (Fig. 4C). Except for strain AL051, from which neither *fimA* nor the invertible element could be PCR-amplified, all strains expressed an approximately14 kDa protein that reacted with antiserum raised against FimA_CFT073_. Band intensity may reflect differences in expression level among strains or, more likely, differences in antibody reactivity to diverse FimA antigens. Indeed, the nucleotide sequences of the *fimA* genes of the clinical isolates are 90–99% identical to *fimA*_CFT073_, with strain AL371 having the highest identity (Fig. S4).

Finally, to assess the assembly of functional fimbriae, the ability of these isolates to agglutinate guinea pig erythrocytes in a mannose-sensitive manner was measured. With the exception again of AL051, all strains exhibited some degree of mannose-sensitive hemagglutination, indicative of type 1 fimbrial production (Fig. 4D). Strain AL371 consistently displayed weak, but detectable hemagglutination. Together, these data demonstrate that, although expression of type 1 fimbrial genes was generally not detected in the urines of cystitis patients, 7 of 8 clinical *E. coli* isolates obtained from women with UTI are capable of appropriately expressing functional type 1 fimbriae *in vitro*.

### Clinical UPEC isolates upregulate *fimA* during murine UTI

**Figure 5:**
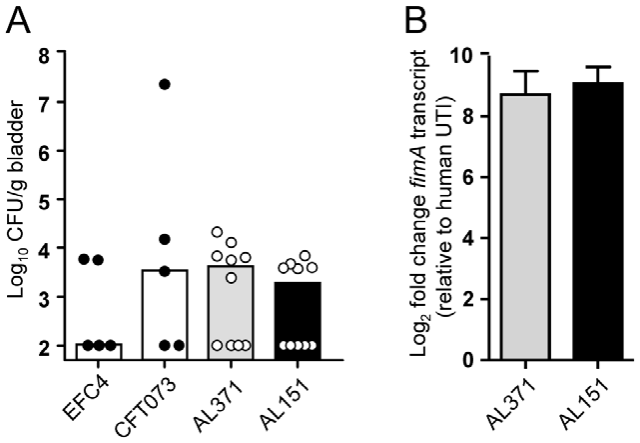
Murine colonization and type 1 fimbriae expression by clinical isolates. CBA/J mice were transurethrally inoculated with 10^8^ CFU *E. coli* EFC4 (fecal strain), CFT073 (model pyelonephritis strain), or clinical isolates AL371 or AL151. (A) Bladder colonization at 48 hpi. Symbols represent data from individual animals and bars indicate the median. (B) *fimA* expression by isolates AL371 (gray bars) and AL151 (black bars) during murine UTI. Urine was collected from infected mice 6–48 hpi and transcript levels from expelled bacteria measured by qPCR. For each strain, urines were pooled from five mice (*n* = 10) and represent two biological replicates. Expression fold change following expulsion from the murine bladder relative to expression following expulsion from the human bladder is shown.

The transcriptome analyses presented in this study identified genes differentially expressed in the urine of different mammalian hosts (Fig. 3). However, these data were obtained by comparing the expression of *E. coli* clinical isolates following collection from human UTI with *E. coli* strain CFT073 expression during murine experimental UTI. As a result, the expression incongruencies could be due to inherent strain-specific differences that exist between the model *E. coli* strain CFT073 and the clinical isolates collected from infected women. To address this, the isolates (except for Fim^−^ strain AL051) were tested in the murine model of ascending UTI. Unlike fecal strain EFC4, which was shown previously to have low infectivity in mice [56], all isolates except AL241 colonized the bladders of CBA/J mice (Fig. 5A and data not shown).

To directly compare the gene expression of a single strain after its colonization of the human and murine urinary tracts, urine was collected and pooled throughout the 48 h infection for strains AL151 and AL371 and transcripts isolated from these pooled samples were quantified by qPCR. In contrast to human infection, both clinical isolates strongly upregulated *fimA* in the urine of infected mice (Fig. 5B). Relative to expression during human UTI, *fimA* was upregulated 660- and 640-fold in the murine bladder by strains AL151 and AL371, respectively. These data, which reflect the change in relative expression rank between murine and human infection (Fig. 3), indicate not only that strains AL151 and AL371 are capable of expressing type 1 fimbriae *in vitro*, but that they robustly express *fimA* during murine UTI. Thus, it appears that these *E. coli* strains had downregulated type 1 fimbriae in the urine of women with cystitis.

## Discussion

Here we report, for the first time, the transcriptional profile for any pathogenic *E. coli* following a naturally-occurring human infection. This study represents the largest number of subjects for any human transcriptome study and includes the largest number of bacterial strains studied in such experiments. Our data suggest that *E. coli* had access to abundant carbon sources prior to expulsion from the human bladder and replicates rapidly, expressing genes involved in nitrogen assimilation, iron acquisition, and virulence, while variably expressing or downregulating at least one major adhesin as assessed in voided urine at the time of sample collection.

Although the women sampled have a history of recurrent UTI, our data suggest that the *E. coli* strains measured in this study represent UPEC strains with full pathogenic potential. All strains were able to colonize a mouse model of ascending UTI, unlike fecal-commensal *E. coli*, and genotype analysis classified them with normal cystitis strains (P. Vigil and H. Mobley, in preparation). All patients in the present study had pyuria, indicating the presence of a robust inflammatory response, which is generally absent in patients with asymptomatic bacteriuria [57]. Nonetheless, it is important to note that the data presented here indeed represent a limited sampling of patients and future studies should expand this analysis to a larger population.

Iron acquisition systems were the most highly expressed virulence determinants across all eight patient isolates *in vivo*. In addition to bacteria isolated from urine, we and other groups have observed expression of outer membrane iron receptors by bladder cell-associated UPEC, both *in vitro* [58] and *in vivo* [51]. Iron acquisition is required for urinary tract colonization [25] and UPEC isolates produce siderophores that are not synthesized by most non-pathogenic fecal *E. coli* strains [59]. Consequently, outer membrane iron receptors have been examined as targets of a vaccine to protect against *E. coli* UTI [60], [61]. Data presented here indicate that at least one protective antigen, Hma, is expressed by at least half of *E. coli* populations during expulsion from the human urinary tract. Of note, the antigen that generated the highest IgA titer, IreA, was not expressed by any patient isolate examined, while the non-protective antigen ChuA was among the most highly expressed genes in all strains. It is interesting to speculate that pressure from the host adaptive immune response may represent negative selection against these genes and/or their regulatory elements.

Despite the iron- and nitrogen-limiting conditions within the human urinary tract, our data support a model of robust UPEC replication during infection. When rapid growth and excess reducing equivalents (NADH and FADH_2_) impose a limitation on the oxidative-dependent TCA cycle, acetate can be excreted to maintain redox homeostasis and recycle coenzyme A [49]. Recently, Welch and colleagues demonstrated that UPEC is better adapted to acetogenic growth than *E. coli* K12 and showed that mutants defective in acetate dissimilation (*pta* and *ackA*) had reduced fitness during murine UTI, while a mutant defective in acetate assimilation (*acs*) did not [47]. Our data support these findings and suggest that UPEC is undergoing similar growth and metabolism during human cystitis. Furthermore, this implies that acetyl-phosphate, which accumulates during acetogenic growth and can act as an intracellular signal, could play a role in UPEC pathogenesis during UTI in women. While we indeed observed differential expression of a number of metabolic genes, flux through these pathways is often regulated posttranscriptionally by enzyme activity and allosteric mechanisms [62], [63], so it is not surprising that a complete metabolic profile cannot be detailed solely from transcriptional data.

Our data suggest that growth of *E. coli* in the human urinary tract is similar to its replication in a chemostat culture. Urine has been described as a mixture of small peptides and amino acids [64] and urine production by the kidneys assures that this medium is continuously replenished. Thus, in contrast to culture in urine *ex vivo*, *E. coli* in the human urinary tract likely has constant access to easily assimilable carbon sources. These results also imply that, although nitrogen and iron are limiting in this environment, *E. coli* acquires adequate quantities of these elements for robust replication.

Whether *E. coli* present in voided urine accurately represent the physiological state of the bacteria attached to and within the bladder mucosa is unclear [65]. Because the majority of UTI pathogenesis occurs on the bladder epithelium, the critical contribution of adherent bacteria is apparent. In contrast, the pathogenic contribution of luminal *E. coli*, which are diagnostic for UTI, is largely undefined. While several groups have measured global gene expression by various *E. coli* strains in urine from infected mice [3], [4] and humans [35], the transcriptome of bladder-associated UPEC has not yet been described and is obviously not feasible in human patients. However, genes identified as highly expressed or upregulated in the urine of infected mice, such as type 1 fimbriae and iron acquisition systems [4], frequently have roles in colonization [25], [28], [66], [67], suggesting that these genes are expressed at some point during association with the murine bladder. Furthermore, qPCR analysis of laser-capture microdissected UPEC from within urothelial cells of infected mice showed increased expression of ferric iron acquisition genes, including the heme receptor *chuA* [51]. These data are consistent with our transcriptome studies of experimental [4] and natural UTI; both identified *chuA* as one of the most highly expressed genes in the urine of infected mice and cystitis patients. Taken together, these studies strongly suggest that, at least in mice, voided urine represents a reasonable estimate of virulence gene expression during cystitis.

Surprisingly, none of the major adherence factors described for UPEC were appreciably expressed in bacteria in urine voided from the human bladder. Because surface structures like fimbriae are known to vary antigenically, sequence dissimilarities between the clinical isolates tested and the UPEC genome represented on the microarray likely contributed at least partially to the low fimbrial detection. Indeed, for type 1 fimbriae, sequence divergence appeared to account for the low detection of the major structural subunit gene *fimA*, as transcript could not be detected for any isolate by microarray, while strains AL231 and AL241 were shown to express this gene by qPCR. However, expression of the remaining *fim* locus was indeed detected for these two patient isolates (AL231 and AL241), indicating that these genes may be more conserved. Similarly, at least four patient isolates encode the major P fimbrial subunit *papA_2* (AL151, AL241, AL361, AL371), but neither microarray nor qPCR could detect *papA_2* transcript in the corresponding *in vivo* samples. Although not critical for virulence in a murine model of infection [68], P fimbriae have long been associated with pyelonephritis in humans [37]. Indeed, *E. coli* that react with anti-P fimbrial antibodies can be isolated from the urine of patients with UTI [69], [70], [71]. The fact that P fimbrial gene expression was not detected in our patient isolates may be due to temporal or localized expression of these genes, or a result of our focus on patients with cystitis, rather than pyelonephritis.

Data from previous studies have implied expression of type 1 fimbriae by a small or variable subset of *E. coli* in human urine. Indirect immunofluorescence of bacteria present in urine of patients with acute UTI has yielded varied results with respect to type 1 fimbrial detection. Several studies identified type 1 fimbriate cells in less than 38% [70] or 45% [72] of urine samples, but nearly in 100% of the same isolates following *in vitro* culture, while another group observed type 1 fimbriate cells in 76% of urine specimens [69]. Experiments from our laboratory quantified the type 1 fimbrial invertible element switch orientation in a bacterial population collected directly from the urine of 11 women with *E. coli* UTI. In that study, the switch was primarily in the “off” position within this population; for all 11 cases, bacteria in patient urine averaged only 4% “on” [73]. While there appears to be some variation among patient populations, these findings are overall consistent with our observation that only 25% of *E. coli* isolates expressed type 1 fimbrial genes in urine collected from cystitis patients.

Several models could account for our finding that the majority of *E. coli* are not transcriptionally active for type 1 fimbriae in urine collected from cystitis patients. First, it is possible that *E. coli* present in voided urine represent the nonadherent “losing” fraction of the population that is expelled from the bladder. However, the samples used in our microarray were not processed to remove exfoliated epithelial cells, so it is likely that both planktonic and adherent bacteria were present to some extent and that neither population significantly expressed type 1 fimbriae. Moreover, measurement of the UPEC transcriptome during murine UTI also relied on the collection of expelled urine from infected animals and those data identified *fimA* as the fourth most highly *in vivo*-expressed gene. Nonetheless, adhesin gene expression differences between adherent and planktonic *E. coli* during human infection likely contributed to our results and should be further examined in future studies.

The duration of infection likely also contributed at least somewhat to our variable detection of fimbriae expression. Urine was collected from mice infected with strains AL151 and AL371 from 6–48 hpi (Fig. 5), while it is unknown how long the women in this study were colonized at the time of sample collection. Although it is well-established that type 1 fimbriae are critical for the establishment of infection in mice [28], [74], their role during bacterial persistence has not been characterized. Consequently, the human urine samples collected in this study may have represented later stages of UTI, during which type 1 fimbrial genes may not be expressed or after fimbriated cells had been cleared by the immune system. In a mouse model of UTI, the orientation of the *fim* invertible element varied throughout the course of infection and by strain, with cystitis strains generally maintaining their IEs in the phase-on position throughout the infection (up to 96 hpi) [75]. Similarly, expression of type 1 fimbriae was shown to be required for UPEC fitness subsequent to the initial attachment/invasion event in a murine model of intracellular replication [76]. Furthermore, transcriptome analysis of UPEC during murine UTI analyzed urine collected up to 10 days post-infection (with a reinfection at day 6) and still observed a high level of *fim* expression [4], so it is unclear whether infection duration alone can explain our results. Nevertheless, future studies should attempt to correlate UTI symptom duration with type 1 fimbrial gene expression of *E. coli* collected from human urine.

Given the abundance of data from our and other laboratories demonstrating the importance of type 1 fimbriae for UTI [27], [28], [29], [77] and positive selection for *fimH* among UTI isolates [16], a likely explanation for our findings is that expression of these genes may be a transient or regulated event during human infection. Analogous to flagellin, which is tightly regulated and only maximally expressed during ascension to the kidneys [19], *fim* expression might be temporally or spatially controlled. We may speculate that, while phase-on bacteria would be primed to adhere to and invade the bladder epithelium, perhaps switching to the phase-off orientation allows dispersal or immune avoidance. Thus, future delineation of the molecular basis for the apparent variable type 1 fimbriae expression detected in our samples, as well as distinction between global gene expression in planktonic *versus* adherent bacteria in urine voided during human UTI will be necessary.

Differences in urinary tract environments among patients and between mammalian hosts are also expected to account for some of the variable expression patterns observed in this study. Diet, hydration, and genetic factors all influence urine composition, urinary tract physiology and, most likely, gene expression by colonizing bacteria. For example, amino acids, temperature [78], sialic acid [79], oxygenation [80], pH and osmolarity [81] are known to affect the orientation of the invertible element region and thus, fimbrial expression. As the present study represents an initial investigation of UPEC gene expression during human UTI, further analysis of additional patient samples will be needed to more completely assess potential correlations between urine chemistry and UPEC gene expression in urine from patients with UTI.

The data presented in this study provide the first insights into pathogenic *E. coli* gene expression within the human host. Our findings are generally consistent with data generated using murine models and support the current model of UPEC pathogenesis. In urine from women with cystitis, *E. coli* express metabolic genes consistent with rapid replication and acetate excretion, actively scavenge iron, express known virulence genes, and may modulate expression of genes involved in motility and adherence. Continued investigation of UPEC gene expression in the urine of UTI patients will contribute both to our understanding of UPEC pathogenesis and to the development of effective UTI therapies.

## Materials and Methods

### Ethics statement

Non-pregnant women over the age of 18 years with symptoms indicative of a UTI were invited to participate in our study by A.L.L. or G.J.F. Written consent was obtained from all subjects prior to enrollment and patient samples were assigned arbitrary identification based on the order of enrollment in our study. All human subject protocols were approved by the Institutional Review Boards of the University of Michigan Medical School (HUM00011155). All animal procedures were conducted in accordance with the guidelines of the University Committee on Use and Care of Animals at the University of Michigan Medical School and following protocols approved by UCUCA.

### Bacterial strains

Clinical *E. coli* isolates were cultured from the urine of women with suspected UTIs using standard methods [82]. Antimicrobial susceptibility testing was performed on all clinical *E. coli* isolates cultured from the urine of the women participating in our study by the Clinical Microbiology Laboratory at the University of Michigan Health System using the VITEK 2 system (bioMerieux, Durham, NC). Clinical isolates were serotyped by the *E. coli* Reference Center at the Pennsylvania State University using antisera against O1-O181 (except O31, O47, O72, O93, O94, and O121, which were not designated) and PCR-restriction fragment length polymorphism analysis of the *fliC* gene.

*E. coli* CFT073 was isolated from the blood and urine of a patient with acute pyelonephritis and *E. coli* EFC4 was isolated from the feces of a healthy woman with no history of a UTI or antibiotic use in the previous six months [56]. *E. coli* K12 is the prototypical commensal strain, MG1655 [83]. CFT073 type 1 fimbriae phase locked-on (L-ON) and locked-off (L-OFF) mutants were constructed as previously described by our laboratory [84]. Strains were routinely cultured in Luria broth (10 g/L tryptone, 5 g/L yeast extract, 0.5 g/L NaCl) at 37°C with aeration, unless otherwise noted.

### Sample collection and *in vivo* RNA isolation

Fresh mid-stream urine was collected from consenting women with presumptive bacteriuria attending the University of Michigan Urology clinic. A diagnosis of presumptive bacteriuria was made based on symptoms of urgency and frequency and/or a history of previous UTI. Volumes collected ranged from 28 to 187 ml, with a median volume of 70 ml (average = 78.8 ml). Urine was collected from 34 women in order to obtain 10 *E. coli*-positive samples that were suitable for our study. Of these, two samples contained multiple *E. coli* strains and were not analyzed further. For the eight patients from whom single strains of *E. coli* were isolated and studied in this report, no patient was catheterized. Seven of eight patients reported a previous UTI. Two patients were taking one antibiotic (ciprofloxacin or nitrofurantoin); however, each respective *E. coli* strain was resistant to that antibiotic.

Collected urine was immediately tested by urinalysis and analyzed by wet-mount microscopy for the presence of bacteria. Specimens positive for leukocyte esterase and/or nitrites, and/or those containing visible bacteria by microscopy were immediately stabilized (within 10 min of sample collection) by the addition of 2 volumes of RNAprotect (Qiagen) and incubated at 25°C for at least 10 min. Stabilized urine specimens were collected by centrifugation (3000×*g*, 30 min, 25°C) and stabilized bacterial pellets were stored at −80°C for up to four weeks. Upon receipt of the clinical culture and sensitivity results, the *E. coli*-positive samples were processed for RNA isolation using the RNeasy Mini system (Qiagen) according to the manufacturer's instructions. DNA was removed from the preparation using TURBO DNase (Ambion) and, where necessary, RNA samples were concentrated using MinElute columns (Qiagen). It is important to note that samples likely also contained human RNA, which was not quantified or removed.

### *In vitro* RNA isolation

Clinical *E. coli* isolates were cultured overnight in LB, washed twice in pooled, filter-sterilized human urine (pooled from 5 healthy donors) and adjusted to OD_600_ = 4.0. Standardized bacterial suspension was inoculated 1∶100 into 25 ml human urine (starting OD_600_ = 0.004) and cultured statically at 37°C until OD_600_ = 0.2±0.02. Culture aliquots (5 ml) were stabilized with 10 ml RNAprotect and total RNA was isolated using the RNeasy Mini procedure described above.

All RNA and cDNA preparations were analyzed using the Agilent 2100 Bioanalyzer (Agilent Technologies) to verify sample quality and integrity. Each sample met the criteria A_260_/A_280_≥1.7 and A_260_/A_230_≥1.5. Concentrations of total RNA and cDNA samples were determined using a NanoDrop ND-1000 spectrophotometer (Thermo Scientific).

### cDNA synthesis, labeling and microarray hybridization

cDNA was synthesized from total RNA isolated using the Superscript Double-Stranded cDNA Synthesis system (Invitrogen) according to the manufacturer's instructions. The only modifications to the protocol were an increase in random primer concentration (3 μg) and extension of the reverse transcriptase reaction (42°C, 90–120 min). cDNA was labeled and hybridized by Roche NimbleGen (Madison, WI) according to their standard protocols. Briefly, cDNA was labeled with Cy3 using the One-Color DNA Labeling protocol (Roche NimbleGen) and 15 μg labeled cDNA was prepared for each sample (10 *in vivo* samples and 12 *in vitro* samples, each in triplicate microarrays). Following hybridization, microarrays were washed and scanned using a GenePix 4000B Scanner (Axon Instruments). Data were extracted and analyzed using the Roche NimbleScan software, which normalizes expression data using quantile normalization [85] and generates gene calls using the Robust Multichip Average algorithm [86].

### *E. coli* CFT073 gene-specific microarray and data normalization

The *E. coli* CFT073 microarray (Roche NimbleGen) contains 14 60-mer perfect match probes (no mismatch) for each of the 5379 open reading frames in the annotated CFT073 genome [14], as well as random probes with similar G+C content. Each array value for the 5379 genes was derived from the automated normalization of 5 replicates of probes printed on each slide. The expression value for each potential gene was obtained by hybridizing triplicate samples to the *E. coli* CFT073 microarray. Biological replicates were not performed on the *in vivo* specimens as patients commenced antibiotic therapy following consultation with G.J.F. and the opportunity to collect multiple urine specimens was not possible, therefore *in vivo* microarrays represent technical replicates.

The median value of the three replicates was obtained for each ORF and was compared to the median value of the random probes on each chip. The median value of the random probes on each array was used as a correction factor for the remaining signal on the chip, normalizing the hybridization and RNA quality of each preparation. The resulting absolute intensity values were transformed into log_2_ values and subsequent analysis utilized these log transformed normalized values to determine the potential expression for any given gene represented on the array. The gene was considered to be expressed if the intensity was four-fold above the value of the randomized control values. It is important to note that, as these isolates are uncharacterized, a lack of hybridization does not always indicate that a gene is not expressed, only that under the conditions examined there was not sufficient signal to indicate expression. Gene absence or genetic divergence between gene sequences of the isolates and gene sequences of the reference strain, *E. coli* CFT073 may also explain poor hybridization. Gene expression heat maps were generated using TreeView 1.60. Microarray data have been deposited in NCBI Gene Expression Omnibus [87] and are accessible through GEO Series accession number GSE24478 (http://www.ncbi.nlm.nih.gov/geo/).

### Invertible element assay

The orientation of the *fim* invertible element was determined as described [73] using the primers listed in Table 5. Clinical isolates and strain CFT073 were inoculated into 5 ml LB, incubated statically at 37°C for 48 h, passaged 1∶100 into fresh medium, and incubated at 37°C for an additional 48 h. Aerated cultures were similarly inoculated and incubated at 37°C for ∼4 h. All cultures were adjusted to OD_600_ = 1.0 and 500 µl was centrifuged (10,000×*g*, 1 min) for western blotting (see below). Standardized culture (50 µl) was added to 50 µl water and boiled 10 min. PCR was performed to amplify the invertible element and 2 µl of crude lysate as template. PCR product was digested with *SnaB*I and separated on a 2% agarose gel.

**Table 5:** Primers used in this study.

Gene	Forward^a^	Reverse	Reference
*gapA*	ACGAAGTTGGTGTTGACGTTGTCG	ATAACCACTTTCTTCGCACCAGCG	[19]
*fliC*	ACAGCCTCTCGCTGATCACTCAA	GCGCTGTTAATACGCAAGCCAGAA	[19]
*fimA*	ACTCTGGCAATCGTTGTTCTGTCG	ATCAACAGAGCCTGCATCAACTGC	This study
*papA2*	ACGGGTGAAATTTGATGGAGCCAC	AATTCGCAACTGCTGAGAAGGCAC	This study
*fyuA*	GCCATTGCTAATGCCCAGACTTCA	AGATGAGACGTTGTTGGCTGATGC	This study
*hma*	ATCGTTCGGCAAGCAACCTTTG	ATGCGGATTTGTTTACGGCCTG	[89]
*sat*	ACAATGTTGTCTCTGGCTGTTGCC	GTGATTGTTACGTCTGTTGCTCCG	This study
*sisA*	CAGAATGCCCCGCGTAAGGC	TTCCTGCAGTATATGGCGTGCCTGT	[24]
*glnA*	TTTGCGCTTCACCGATACCAAAGG	TTAATGCCTTTCCAGCCGCCAATC	This study
*gdhA*	TCCTCGGCTTCGAACAAACCTTCA	GGCAGAAACGCATAACTTCGCCTT	This study
*ackA*	AATGTTTCCACCTGCCCGAA	AAAGTTGAGCGCTTCGCTGTGA	This study
*acs*	TAGCTATAAAGAGCTGCACCGCGA	GCTTCCGGTACCATCGGCATATAA	This study
*rplQ*	TATGGCAGGTTCACTGGTTCGTCA	TGGCAAGAGTAATCAGCGGCTCAA	This study
*msbB*	ATCGCTTTAACGCCGCCAAAGTTC	TACGTTCTGGAAAGCAGAGCGACA	This study
*trkA*	GTGCGCGATGCCGATAAGCTATTT	TCAGCGAAGTTCACCACCTGCAAT	This study
*fim* IE^b^	AGTAATGCTGCTCGTTTTGC	GACAGAGCCGACAGAACAAC	[73]

a All primers are listed 5′→3′.

b Type 1 fimbrial invertible element.

### Antisera production and western blotting

The *fimA* gene was cloned into the commercially-available expression vector pBAD-*myc*-HisA (Invitrogen) in-frame with a C-terminal His_6_ tag. Expression was induced by addition of L-arabinose to 100 µM and recombinant protein was isolated on nickel-nitriloacetic acid-agarose columns (Qiagen). Antibodies were raised in rabbits against recombinant FimA-His_6_ excised from SDS-PAGE gels by Rockland Immunochemicals, Inc. (Gilbertsville, PA).

Bacterial pellets collected as described above were resuspended 100 µl acidified water (pH 1.8) and boiled for 10 min. Lysate was mixed with 20 µl 6× SDS-PAGE loading buffer, neutralized with 1 N NaOH, and separated on 15% SDS-PAGE gels. Proteins were transferred to PVDF membrane and blocked with 5% milk in TBS +0.01% Tween-20. FimA was detected with rabbit anti-FimA (1∶2000), followed by anti-rabbit-HRP (1∶25,000) and ECL Plus detection reagents (GE Healthcare).

### Hemagglutination

Strains were passaged statically or cultured with aeration as described above and adjusted to OD_600_ = 0.8. One ml of culture was pelleted (2500×*g*, 2 min, 25°C) and resuspended in 200 µl PBS and serial dilutions of this bacterial suspension (25 µl) were added to the wells of a microtiter plate. A 3% suspension of PBS-washed guinea pig erythrocytes (v/v) was prepared in PBS on ice and 25 µl added to each well. For testing mannose sensitivity, erythrocyte suspension mixed with α-methyl mannoside (Sigma) at 1 mg/ml was added to wells containing undiluted bacterial suspension. Plates were gently rocked and incubated at 25°C for 30–45 min. Hemagglutination titer was defined as the highest dilution of bacterial suspension that yielded a positive reaction.

### Mouse model of ascending UTI

Six- to eight-week female CBA/J mice were transurethrally inoculated as previously described [34]. Clinical isolates were cultured overnight in LB, collected by centrifugation (3000×*g*, 30 min, 25°C) and resuspended in PBS to 2×10^9^ CFU/ml. Bacterial suspension (50 µl/mouse) was delivered directly into the bladders of anesthetized mice via a sterile 0.28 mm inner diameter polyethylene catheter connected to an infusion pump (Harvard Apparatus), with a total inoculum of 1×10^8^ CFU/mouse. At two-hour intervals beginning at 5 h post-inoculation, urine was collected and pooled from each cage of animals (5 mice). Immediately after collection, cold 5% phenol-ethanol stop solution was added, samples centrifuged (10,000×*g*, 1 min, 4°C), and pellets stored at −80°C for RNA isolation. For CFU determination, mice were sacrificed at 48 hpi and urinary tract organs homogenized with a GLH homogenizer (Omni International) in 3 ml PBS. Homogenate was plated on LB agar using an Autoplate 4000 spiral plater (Spiral Biotech) and colonies enumerated with a QCount plate reader (Spiral Biotech). Significance was determined using the two-tailed Mann-Whitney test.

### qPCR

For microarray validation, real-time qPCR was performed with cDNA isolated above for microarray hybridization. Reactions were conducted in a Mx300P instrument (Stratagene), using 30 ng cDNA template, 0.1 µM primers (Table 1), and Brilliant SYBR Green reagents (Stratagene). Data were normalized to *gapA* and analyzed with MxPro 4.0 software (Stratagene). For *fimA* measurement at room temperature, CFT073 wildtype and L-ON were cultured in 70 ml pooled, filter-sterilized human urine for 6 h at 37°C. Cultures were decanted into 120 ml urine collection cups with lids and incubated at room temperature for 60 min. At intervals, 1 ml culture aliquots were mixed with 125 µl cold 5% phenol-ethanol stop solution, centrifuged (10,000×*g*, 1 min, 4°C), and stabilized pellets stored at −80°C. Thawed pellets were resuspended in 100 µl 1 mg/ml lysozyme in TE and RNA isolated using the RNeasy protocol as described above. cDNA was synthesized using SuperScriptII First-Strand Synthesis reagents according to the manufacturer's instructions and qPCR was performed as described.

## Supporting Information

Table S1 Serotyping of clinical *E. coli* strains isolated from the urine of women with presumptive UTIs.
(0.03 MB DOC)

Table S2 Antibiotic resistance profiles of clinical *E. coli* strains isolated from the urine of women with presumptive UTIs.
(0.03 MB DOC)

Table S3 Gene expression summary for clinical *E. coli* strains during *in vivo* and *in vitro* growth.
(0.03 MB DOC)

Table S4 Hypothetical genes downregulated *in vivo* compared to growth in human urine *in vitro*.
(0.06 MB DOC)

Figure S1 UPEC expression of *fimA* following urine culture transition to room temperature. Wildtype (white bars) or *fim* Locked-ON (gray bars) CFT073 were cultured statically for ∼6 h in 70 ml pooled human urine at 37°C. Cultures were decanted into sterile 120 ml urine collection cups with lids and incubated at room temperature. At the indicated timepoints, culture aliquots were stabilized as described in the Methods and expression of *fimA* was measured by qPCR (normalized to *gapA*). Mean relative expression at each timepoint is shown relative to T_0_ and represents three biological replicates from two independent experiments. The right y-axis (black symbols) shows actual urine temperature following transfer from 37°C to room temperature conditions.
(0.07 MB TIF)

Figure S2 Growth of clinical isolates under iron-limiting conditions. Strains were iron-limited overnight in LB with 200 μM 2′2-dipyridyl (DIP), washed in PBS, and inoculated 1∶100 into fresh (A) LB or LB containing (B) 200 μM or (C) 400 μM DIP. Growth curves at 37°C are shown for CFT073 (black circles), K12 (black triangles), AL051 (red circles), AL121 (red triangles), AL151 (blue circles), AL231 (blue triangles), AL241 (green circles), AL291 (green triangles), AL361 (gray circles), and AL371 (gray triangles).
(0.18 MB TIF)

Figure S3 *In vivo* and *in vitro* expression of genes in the yersiniabactin locus. Heat map indicates normalized microarray signal intensities for genes encoding proteins involved in yersiniabactin synthesis and transport in eight *E. coli* isolates during UTI in patients (P) or culture in urine *ex vivo* (C). For reference, the overall most (*rpsK*, +) and least (*pheM*, −) expressed genes are shown in the bottom panels, representing average signal intensities of 15.821 and 3.881, respectively.
(0.15 MB TIF)

Figure S4 Sequence alignment of *fimA* and the invertible element region. Sequences were aligned by ClustalW algorithm using GraphPad Megalign software. ORFs (*fimE* and *fimA*) are boxed and the left and right inverted repeat regions (IRL and IRR) of the invertible element are in blue. Nucleotides differing from CFT073 are shaded and percent nucleotide identities to CFT073 for each *fimA* ORF are indicated at the end of the sequence.
(1.07 MB TIF)
