# Supplementary material for: Escherichia coli Global Gene Expression in Urine from Women with Urinary Tract Infection
Source: PLoS Pathog. 2010 Nov 11;6(11):e1001187. doi: 10.1371/journal.ppat.1001187 (PMC2978726; doi:10.1371/journal.ppat.1001187)
Supplement: Table S1 — Serotyping of clinical E. coli strains isolated from the urine of women with presumptive UTIs. (0.03 MB DOC) [file ppat.1001187.s001.doc]

Table S1. Serotyping of clinical *E. coli* strains isolated from the urine of women with presumptive UTIs .

| Isolate | O type | H type |
| --- | --- | --- |
| AL051 | 119w | 48 |
| AL061*^a^* | 119w | − |
| AL062*^a^* | 160 | − |
| AL121*^b^* | 25 | 4 |
| AL151 | 25 | 4 |
| AL161*^c^* | 6 | 1 |
| AL162*^c^* | 6 | 1 |
| AL231 | 6w | 31 |
| AL241 | − | 4 |
| AL291 | 6 | 1 |
| AL361*^b^* | 25 | 4 |
| AL371 | 134 | 4 |

w, weak reaction; -, no reaction

*^a^* Mixed-strain infection, AL061/AL062 were isolated from the same patient on the same day.

*^b^* Isolated from the same patient on different days.

*^c^* Mixed-strain infection, AL161/AL162 were isolated from the same patient on the same day.
