# Supplementary material for: Escherichia coli Global Gene Expression in Urine from Women with Urinary Tract Infection
Source: PLoS Pathog. 2010 Nov 11;6(11):e1001187. doi: 10.1371/journal.ppat.1001187 (PMC2978726; doi:10.1371/journal.ppat.1001187)
Supplement: Table S2 — Antibiotic resistance profiles of clinical E. coli strains isolated from the urine of women with presumptive UTIs. (0.03 MB DOC) [file ppat.1001187.s002.doc]

Table S2. Antibiotic resistance profiles of clinical *E. coli* strains isolated from the urine of women with presumptive UTIs.

|  | UM Health System*^a^* | | | |  | Study participants*^b^* | | | |
| --- | --- | --- | --- | --- | --- | --- | --- | --- | --- |
| Antibiotic |  | % isolates | | |  |  | % isolates | | |
|  | *n* | R | I | S |  | *n* | R | I | S |
| Ampicillin | 1093 | 49 | 2 | 49 |  | 12 | 57 | 0 | 42 |
| Cefazolin | 1091 | 12 | 4 | 84 |  | 12 | 17 | 0 | 83 |
| Trimeth/Sulfa | 1089 | 26 | ND*^c^* | 74 |  | 12 | 58 | ND | 42 |
| Nitrofurantoin | 1066 | 2 | 4 | 94 |  | 12 | 17 | 0 | 83 |
| Ciprofloxacin | 1083 | 25 | 1 | 74 |  | 12 | 42 | 0 | 58 |
| Levofloxacin | 1082 | 25 | 1 | 74 |  | 12 | 42 | 0 | 58 |

R, resistant; I, intermediate; S, susceptible

*^a^* Data collected from the University of Michigan Health System public database. Values shown are the result of averaging available data from July-Dec 2007 (*n*=582-587) and Jan-June 2008 (*n*=494-508). These data include the isolates collected from study participants.

*^b^* Represents data from all 12 isolates collected in this study, including two mixed-strain infections (accounting for four isolates).

*^c^* Intermediate classification not determined for trimethoprim-sulfamethoxazole.
