# Supplementary material for: Escherichia coli Global Gene Expression in Urine from Women with Urinary Tract Infection
Source: PLoS Pathog. 2010 Nov 11;6(11):e1001187. doi: 10.1371/journal.ppat.1001187 (PMC2978726; doi:10.1371/journal.ppat.1001187)
Supplement: Table S3 — Gene expression summary for clinical E. coli strains during in vivo and in vitro growth. (0.03 MB DOC) [file ppat.1001187.s003.doc]

Table S3. Gene expression summary for clinical *E. coli* strains during *in vivo* and *in vitro* growth.

|  | No. genes expressed*^a^* | |
| --- | --- | --- |
| Isolate | *in vivo* | *in vitro^b^* |
| AL051 | 1137 | 1348 |
| AL121 | 1788 | 1227 |
| AL151 | 1870 | 1084 |
| AL231 | 1472 | 1296 |
| AL241 | 317 | 1165 |
| AL291 | 1711 | 1447 |
| AL361 | 2044 | 1381 |
| AL371 | 1830 | 1436 |

*^a^* Expression defined as microarray hybridization intensity at least four-fold above background.

*^b^* During culture in pooled human urine *ex vivo*.
