# Supplementary material for: Escherichia coli Global Gene Expression in Urine from Women with Urinary Tract Infection
Source: PLoS Pathog. 2010 Nov 11;6(11):e1001187. doi: 10.1371/journal.ppat.1001187 (PMC2978726; doi:10.1371/journal.ppat.1001187)
Supplement: Table S4 — Hypothetical genes downregulated in vivo compared to growth in human urine in vitro. (0.06 MB DOC) [file ppat.1001187.s004.doc]

Table S4. Hypothetical genes downregulated *in vivo* compared to growth in human urine *in vitro*.

| Gene | Annotation | Median  Δ rank*^a^* | No. strains*^b^* (*n*=8) |
| --- | --- | --- | --- |
| *yeaG* | Hypothetical protein | -1714 | 8 |
| *yceK* | Hypothetical protein precursor | -1705 | 8 |
| *ygaT* | Hypothetical protein | -1694 | 8 |
| c5459 | Conserved hypothetical protein | -1680 | 8 |
| *ygaU* | Unknown protein from 2D-page | -1629 | 8 |
| *yegP* | Hypothetical protein | -1614 | 8 |
| *yccJ* | Hypothetical protein | -1613 | 8 |
| *ycgB* | Hypothetical protein | -1609 | 8 |
| c1317 | Hypothetical protein | -1576 | 8 |
| *yjcH* | Hypothetical protein | -1530 | 8 |
| *ygaM* | Hypothetical protein | -1529 | 8 |
| c5458 | Hypothetical protein | -1527 | 8 |
| c0088 | Hypothetical protein | -1513 | 7 |
| *ygaF* | Hypothetical protein | -1434 | 7 |
| *yohC* | Hypothetical protein | -1427 | 7 |
| *ybeL* | Hypothetical protein | -1401 | 8 |
| *ychH* | Hypothetical protein | -1387 | 7 |
| *yhjY* | Hypothetical protein | -1373 | 7 |
| c2122 | Hypothetical protein | -1342 | 7 |
| *ydcJ* | Hypothetical protein | -1305 | 8 |
| *yoaC* | Hypothetical protein | -1301 | 7 |
| *yccD* | Hypothetical protein | -1295 | 7 |
| *yeaQ* | Hypothetical protein | -1291 | 7 |
| *yidB* | Hypothetical protein | -1286 | 7 |
| *ynaF* | Unknown protein 2D_000B3L from 2D-page | -1282 | 7 |
| c0797 | Hypothetical protein | -1265 | 8 |
| *yiiT* | Hypothetical protein | -1232 | 7 |
| *yjdJ* | Hypothetical protein | -1232 | 7 |
| c5135 | Hypothetical protein | -1127 | 7 |
| *yniA* | Hypothetical protein | -1089 | 7 |
| c3486 | Hypothetical protein | -1084 | 7 |

*^a^* Median change in relative expression rank (genes ranked in order of expression; highest, 1; lowest, 5379) *in vitro* compared to *in vivo*.

*^b^* Number of strains for which indicated gene was among the 250 (4.6%) most downregulated genes. Gene may be downregulated by other strains, but was not among the top 4.6%.
