# Supplementary figures and images for: Escherichia coli Global Gene Expression in Urine from Women with Urinary Tract Infection
Source: PLoS Pathog. 2010 Nov 11;6(11):e1001187. doi: 10.1371/journal.ppat.1001187 (PMC2978726; doi:10.1371/journal.ppat.1001187)

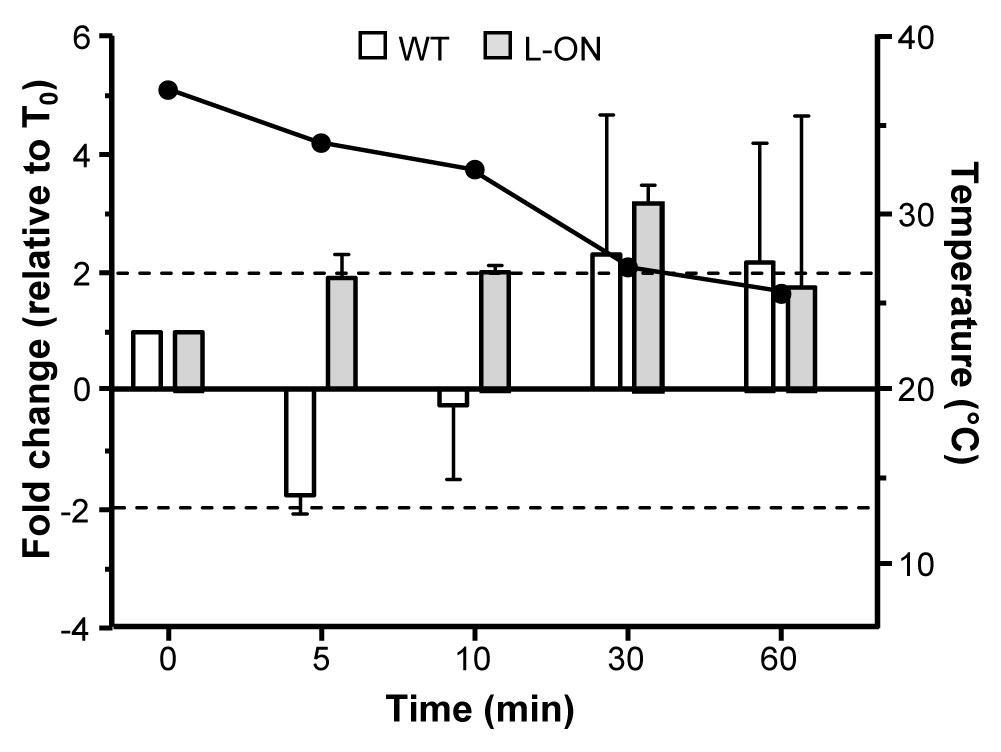

Supplement: Figure S1 — UPEC expression of fimA following urine culture transition to room temperature. Wildtype (white bars) or fim Locked-ON (gray bars) CFT073 were cultured statically for ∼6 h in 70 ml pooled human urine at 37°C. Cultures were decanted into sterile 120 ml urine collection cups with lids and incubated at room temperature. At the indicated timepoints, culture aliquots were stabilized as described in the Methods and expression of fimA was measured by qPCR (normalized to gapA). Mean relative expression at each timepoint is shown relative to T0 and represents three biological replicates from two independent experiments. The right y-axis (black symbols) shows actual urine temperature following transfer from 37°C to room temperature conditions. (0.07 MB TIF) [file ppat.1001187.s005.tif]

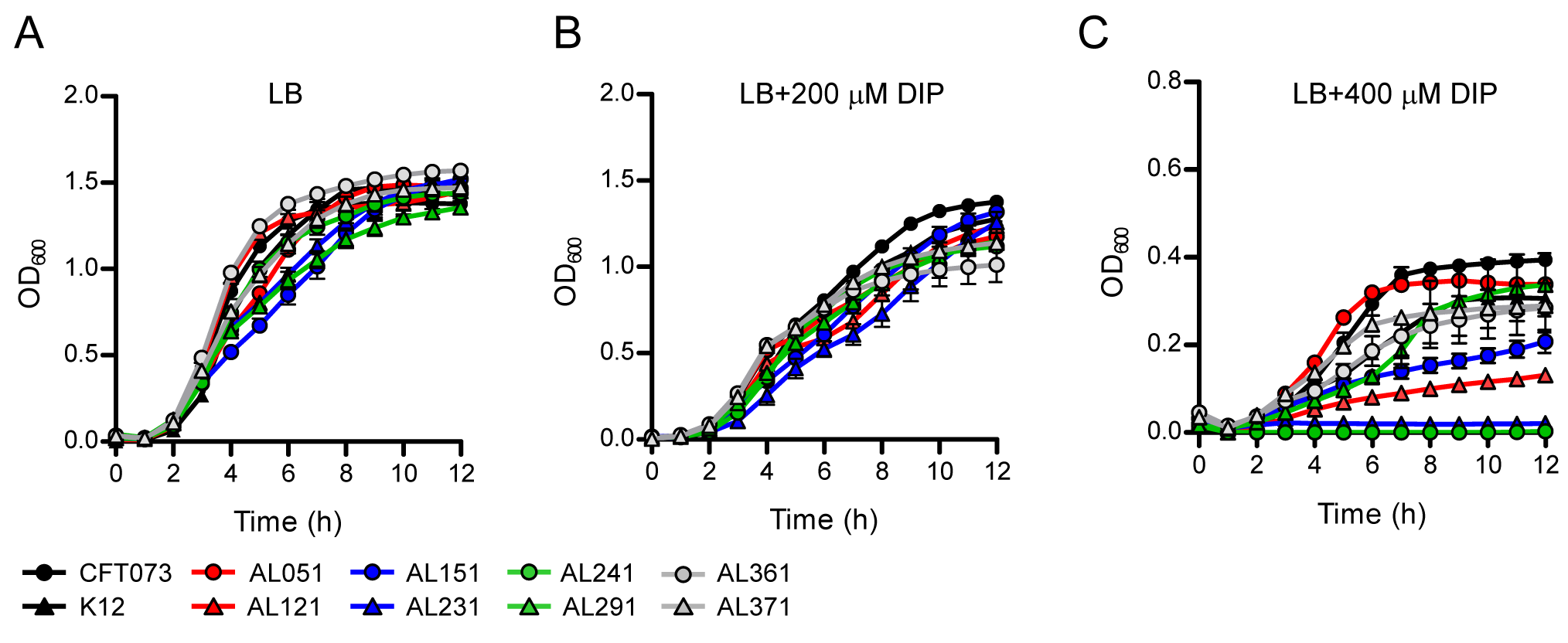

Supplement: Figure S2 — Growth of clinical isolates under iron-limiting conditions. Strains were iron-limited overnight in LB with 200 μM 2′2-dipyridyl (DIP), washed in PBS, and inoculated 1∶100 into fresh (A) LB or LB containing (B) 200 μM or (C) 400 μM DIP. Growth curves at 37°C are shown for CFT073 (black circles), K12 (black triangles), AL051 (red circles), AL121 (red triangles), AL151 (blue circles), AL231 (blue triangles), AL241 (green circles), AL291 (green triangles), AL361 (gray circles), and AL371 (gray triangles). (0.18 MB TIF) [file ppat.1001187.s006.tif]

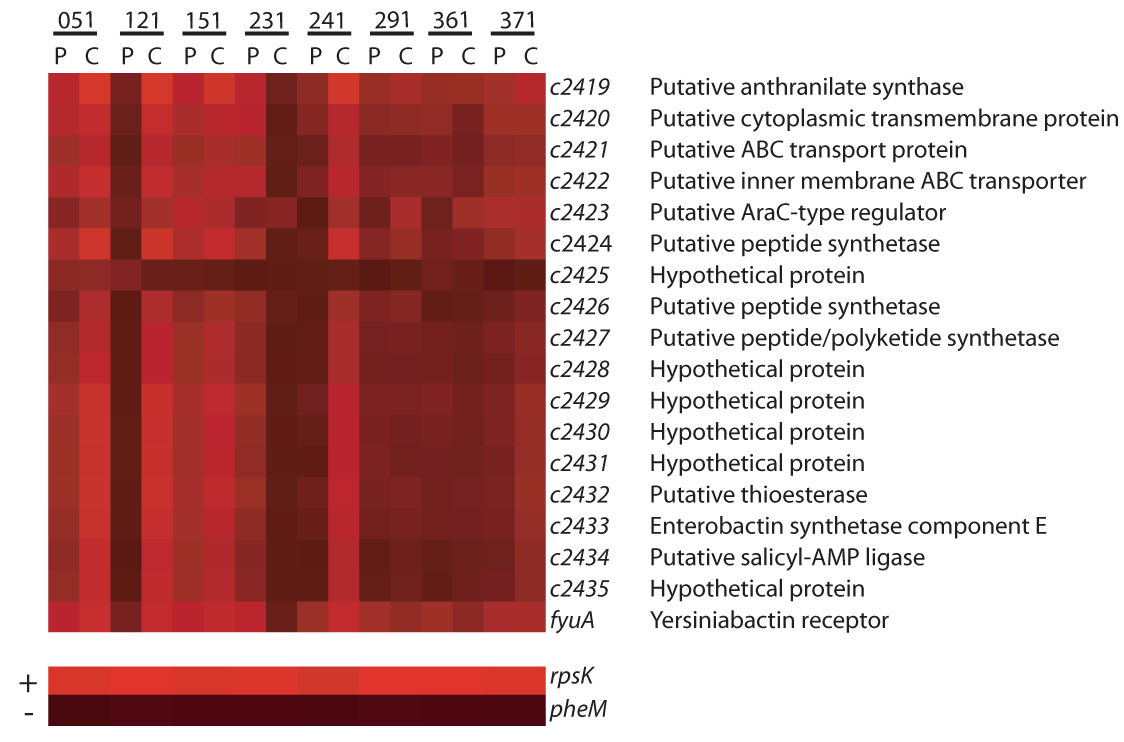

Supplement: Figure S3 — In vivo and in vitro expression of genes in the yersiniabactin locus. Heat map indicates normalized microarray signal intensities for genes encoding proteins involved in yersiniabactin synthesis and transport in eight E. coli isolates during UTI in patients (P) or culture in urine ex vivo (C). For reference, the overall most (rpsK, +) and least (pheM, −) expressed genes are shown in the bottom panels, representing average signal intensities of 15.821 and 3.881, respectively. (0.15 MB TIF) [file ppat.1001187.s007.tif]

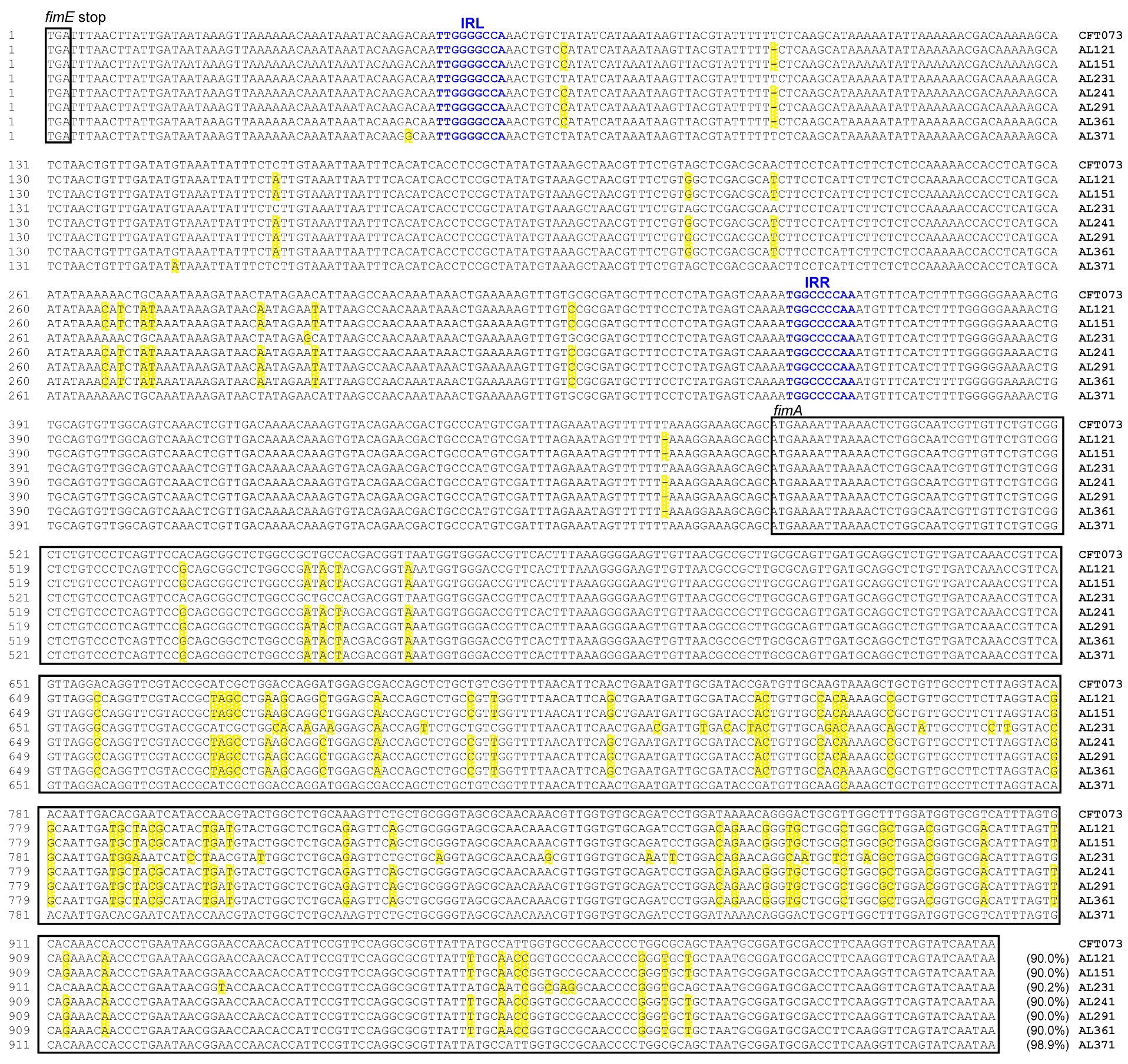

Supplement: Figure S4 — Sequence alignment of fimA and the invertible element region. Sequences were aligned by ClustalW algorithm using GraphPad Megalign software. ORFs (fimE and fimA) are boxed and the left and right inverted repeat regions (IRL and IRR) of the invertible element are in blue. Nucleotides differing from CFT073 are shaded and percent nucleotide identities to CFT073 for each fimA ORF are indicated at the end of the sequence. (1.07 MB TIF) [file ppat.1001187.s008.tif]
